# Pharmacological inhibition of S6K1 rescues synaptic deficits and attenuates seizures and depression in chronic epileptic rats

**DOI:** 10.1111/cns.14475

**Published:** 2023-09-22

**Authors:** Yuying Zhang, Xiaojuan Cheng, Luyan Wu, Juan Li, Changyun Liu, Mingjia Wei, Chaofeng Zhu, Huapin Huang, Wanhui Lin

**Affiliations:** ^1^ Fujian Medical University Union Hospital Fuzhou China; ^2^ Fujian Key Laboratory of Molecular Neurology Fujian Medical University Fuzhou China; ^3^ Fujian Medical University Second Affiliated Hospital Quanzhou China

**Keywords:** anxiety and depression, epilepsy, local field potentiation, long‐term potentiation, S6K1, synaptic plasticity, theta and delta power

## Abstract

**Background:**

Recent studies have shown that mTOR signaling plays an important role in synaptic plasticity. However, the function of S6K1, the mechanistic target of rapamycin kinase complex 1 (mTORC1) substrate, in epilepsy remains unknown.

**Aims:**

Our present study aimed to explore the mechanism by which S6K1 is involved in chronic epilepsy.

**Methods:**

First, immunostaining was used to measure neurite length and complexity in kainic acid (KA)‐treated primary cultured neurons treated with PF‐4708671, a highly selective S6K1 inhibitor. We obtained evidence for the role of S6K1 in protecting and promoting neuronal growth and development in vitro. Next, to explore the function and mechanism of the S6K1 inhibitor in epilepsy, a pilocarpine‐induced chronic epileptic rat model was established. In vivo electrophysiology (including local field potentiation in CA1 and long‐term potentiation), depression/anxiety‐like behavior tests, and Golgi staining were performed to assess seizure behavior, power spectral density, depression/anxiety‐like behavior, and synaptic plasticity. Furthermore, western blotting was applied to explore the potential molecular mechanisms.

**Results:**

We found that inhibition of S6K1 expression significantly decreased seizures and depression‐like behavior and restored power at low frequencies (1–80 Hz), especially in the delta, theta, and alpha bands, in chronic epileptic rats. In addition, PF‐4708671 reversed the LTP defect in hippocampal CA3–CA1 and corrected spine loss and dendritic pathology.

**Conclusion:**

In conclusion, our data suggest that inhibition of S6K1 attenuates seizures and depression in chronic epileptic rats via the rescue of synaptic structural and functional deficits. Given the wide range of physiological functions of mTOR, inhibition of its effective but relatively simple functional downstream molecules is a promising target for the development of drugs for epilepsy.

## Graphical abstract

Our study aimed to explore the mechanisms of S6K1 involvement in chronic epilepsy. Pharmacological inhibition of S6K1 by in vivo and in vitro levels in an epilepsy model showed that it attenuates seizures and depression in rats with chronic epilepsy by rescuing synaptic structural and functional defects.

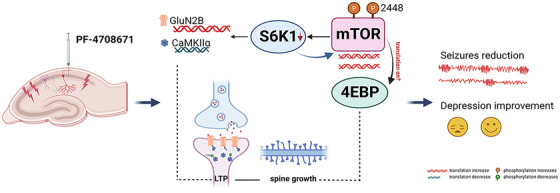

## 1 INTRODUCTION

Epilepsy is one of the most common neurological disorders characterized by a lasting predisposition to generate spontaneous epileptic seizures and has numerous neurobiological, cognitive, and psychosocial consequences, affecting more than 70 million people worldwide.^1^ Depression/anxiety disorders are the most common psychiatric comorbidities. A meta‐analysis showed that the prevalence of depression was as high as 22%–25% in a community‐based study of patients with epilepsy.^2^ Increasing evidence shows the bidirectional effects between epilepsy and depression/anxiety, where two conditions can each contribute to the other.^3^ Neurons have been the focus of epilepsy research and can produce abnormal neural circuits and functional changes through synapse formation. It is reasonable to envision epilepsy as a disease that results from impaired synaptic structure and/or function.^4^ One leading hypothesis for the development and progression of epilepsy is that large‐scale changes in gene and protein expression that control neurotransmitter signaling, synaptic structure, ion channels, cell death, gliosis and inflammation contribute to aberrant network restructuring and hyperexcitability.^5^ Distinct from somatic translation, overwhelming evidence suggests that translation can occur at synapses, and activity‐dependent local translation of many synaptic proteins has been identified as essential for many forms of synaptic plasticity.^6^ It alters the local cellular composition, connectivity, and morphology in response to neuronal activity, leading to long‐term or permanent changes in both synaptogenesis and synaptic excitability, such as long‐term potentiation (LTP) and long‐term depression (LTD), which helps to explain why mutations in translational components affect epilepsy so profoundly.^7^

The mammalian target of rapamycin (mTOR) was identified as a core kinase, and mTOR‐dependent local translation was shown to be critical in molecular studies of neuronal plasticity involved in the regulation of epilepsy.^8^ p70 ribosomal S6 kinase 1 (S6K1), as an extensively studied downstream effector of mTORC1 signaling, is directly involved in the regulation of translation initiation and elongation: by phosphorylating ribosomal protein S6 (rpS6), it recruits phosphorylated eIF4B to eIF4A at the translation initiation complex or phosphorylates PDCD4 to promote its degradation, ultimately increasing the helicase activity of eIF4A, promoting translation elongation by phosphorylation inactivation of eukaryotic elongation factor 2 kinase (eEF2K), and enhancing the translation efficiency of spliced mRNAs via interaction with SKAR.^9^, ^10^ Previous studies on mutations in the tuberous sclerosis complex (TSC), with epilepsy as the main clinical manifestation, have confirmed that mTORC1 is selectively upregulated, and S6Ks (S6K1 and S6K2) are the only AGC kinases activated in this disease.^11^ It has also been suggested that RPS6 is a potential novel intractable epileptic hemimegalencephaly (HME)‐related gene,^12^ but the specific effects on this pathway after seizures remain poorly understood. In addition, the S6K1 inhibitor PF‐4708671 can normalize hippocampal LTP by promoting actin polymerization. It was previously reported that actin dynamics regulate AMPA receptor trafficking during synaptic plasticity and reverse the decrease in GluA1 levels in the hippocampus, which was shown to be dysregulated in an epilepsy model.^13^, ^14^ Therefore, we hypothesize that inhibition of S6K1 activity in chronic epileptic rats may modulate local translation and affect synaptic function and morphology, thereby correcting the multiple phenotypes displayed by epileptic rats.

To test this hypothesis, we used PF‐4708671, a highly selective S6K1 inhibitor, to pharmacologically inhibit S6K1 in both KA‐treated primary cultured neurons and chronic epileptic rats. We found that injured neurites were protected. In addition, defective CA3–CA1 LTP and immature spines were rescued. Several behavioral abnormalities exhibited by the epileptic model rat, including increased seizures and depression, were prevented. This improvement in synaptic plasticity was accompanied by behavioral recovery, suggesting that synaptic function and structural remodeling induced by S6K1 inhibition may help correct the abnormal phenotype of epilepsy. Thus, S6K1 may be a potential target for therapeutic intervention in patients with epilepsy.

## 2 MATERIALS AND METHODS

### 2.1 Animals and ethics approval

All experimental animals were adult male (200–250 g) Sprague–Dawley rats from the Experimental Animal Center of Fujian Medical University, with four or five per R5 rat herd cage (size: 545 × 395 × 200 mm) in an SPF animal laboratory (at 22–24°C under a 12/12 light–dark cycle, light on at 7:00 a.m.; food and water available ad libitum).

All animal experiments were performed following the guidelines set by the Institutional Animal Care and Use Committee of Fujian Medical University.

### 2.2 Establishment of the status epilepticus (SE) model in rats

As previously described, status epilepticus (SE) was induced by the administration of lithium‐pilocarpine following our previous study (see Supporting Information).^15^ Repeated doses (≤3) of pilocarpine hydrochloride (30 mg/kg, i.p., Sigma–Aldrich, P6503) were given to the rat every 30 min until the emergence of SE (Stage IV–V on Racine's scale).^16^ More than 60 min after the onset of SE, diazepam (10 mg/kg, i.p.) was administered to terminate the ictal activity.

All pilocarpine‐treated rats included in this study showed an onset of seizure activity within 60 min after pilocarpine administration and developed SE.

### 2.3 Hippocampal injection of PF‐4708671

Operated rats for PF‐4708671 (Selleck, S2163) injections were induced with 5% isoflurane anesthesia (RWD Life Sciences) in 100% O_2_ for 2 min, and then anesthesia was maintained at isoflurane concentrations of 2% using a stereotaxic instrument (RWD Life Sciences). Briefly, PF‐4708671 injections were performed as described.^17^ One microlitre/injection site of PF‐4708671 was delivered to the CA1 area of the hippocampus using a 5‐μL syringe (Hamilton, Model 75N). The injection sites were identified from the rat stereotaxic atlas of Paxinos and Watson (see Supporting Information). After at least a 7‐day recovery period, animals were allowed to proceed to the next experiment.

### 2.4 Electrode implantation

Rats were mounted in a stereotaxic instrument under isoflurane anesthesia as described previously. The hole was drilled first through the skull, and electroencephalogram and electromyogram (EMG) electrodes were implanted. For details, see the Supporting Information. The assembly was fixed to the skull using dental cement. After surgery, rats were kept warm, received saline (0.9% NaCl, s.c.) and were housed individually after recovery from anesthesia. Rats were allowed to recover for at least 10 days before their adaptation to the custom‐made transparent cage (30 cm × 30 cm × 40 cm) and recording cable through a slip ring for one day.

### 2.5 LFP/EMG recordings

Ten or more days after surgery and custom‐made transparent cage adaptation for one day, the LFP and EMG signals were amplified, filtered (LFP 0.5–1000 Hz; EMG, 10–500 Hz), digitized (at a sampling frequency of 2000/s) and recorded for 12 h using a PowerLab8/35 system (ADInstruments) with synchronized video recording. All the experimental data were analyzed offline using Lab Chart Acquisition software (Pinnacle Technologies) and MATLAB (Math Works). All the waveforms were classified by a trained observer.^18^

### 2.6 Analysis of LFP data

As previously described,^19^ the power spectral density (PSD) values in 7 frequency bands were calculated by fast Fourier transform (FFT) using MATLAB software (Math Works). To balance individual variability, we randomly selected three nonepileptiform discharge LFP segments as a baseline of approximately equal duration to the mean epileptiform discharge duration for each rat in each group to normalize the PSD values of LFP power in different frequency bands for each rat.

### 2.7 In vivo electrophysiology

Rats were mounted in a stereotaxic instrument under isoflurane anesthesia as described previously. A tungsten recording microelectrode (127 μm in diameter) was implanted in the hippocampal CA1 region as described in the electrode implantation method. A bipolar platinum‐Iridium wire stimulating electrode (250 μm outer diameter with a 100 μm tip separation; KD) was placed in the CA3 region of the hippocampus (AP: 4.5 mm, ML: 4.0 mm, DV: 3.65 ± 0.2 mm, following Paxinos and Watson, 2005) ipsilateral to the recording site. The final depth of the stimulating and recording electrodes was at the site where the maximum amplitude of the field excitatory postsynaptic potential (fEPSP) is evoked on the CA1 recording electrode. Evoked fEPSPs were amplified, filtered (0.5–1 kHz), digitized (at a sampling frequency of 2 kHz), and stored on an Off‐line computer using the PowerLab8/35 system. The fEPSP amplitude was determined with a PowerLab8/35 system. The evoked frequency of the test EPSPs was 0.01 Hz, and the stimulus intensity was adjusted to 40%–60% of the maximum fEPSP amplitude. After recording stable baseline data for 30 min (1 stimulation every 10 s, 0.12 ms duration pulses), the high frequency stimulation protocol for inducing LTP was composed of 4 series of 10 trains (200 Hz, 0.12 ms) at 0.1 Hz was delivered to the CA1 at the test intensity. Percent of the baseline fEPSP amplitude recorded over at least a 30‐min baseline period.

### 2.8 Behavioral tests

Behavioral tests targeted depression‐anxiety‐like behaviors, including the sugar water test, forced swim test (FST), tail suspension test (TST), and elevated plus maze (EPM). All the tests were performed in conditions and in a manner as described previously.^20^, ^21^, ^22^, ^23^ For details, see the Supporting Information.

### 2.9 Protein extraction and Western blotting

Synaptosome protein samples were extracted following the manufacturer's protocols (Syn‐PER™ Synaptic Protein Extraction Reagent, 87793; Thermo Fisher). Equal amounts of protein samples were loaded and separated by 4%–12% SDS–PAGE (GenScript ExpressPlus™ PAGE Gel, M00653) and transferred to PVDF membranes (Millipore). Membranes were incubated with primary antibody overnight at 4°C. After washing, the membranes were incubated with peroxidase‐conjugated secondary antibodies (1:5000, goat anti‐rabbit IgG, goat anti‐mouse IgG, Abcam) for 1.5 h at room temperature. The immunoreactive bands were visualized by chemiluminescent HRP substrate (WesternBright ECL HRP Substrate Kit, K‐12045‐D50‐EA, Advansta) with a Fluorchem E Chemiluminescence Gel Imaging System (Protein Simple). The intensity of the immunoreactive different exposure times for the same strip was determined with ImageJ software. The expression levels of the proteins of interest were examined as GAPDH/total protein in a semiquantitative manner (see Supporting Information).

### 2.10 Primary hippocampal neuronal cell culture

The hippocampal neuronal cell culture methods employed were similar to those described previously.^24^, ^25^ In the present study, hippocampal neurons maintained in culture for 12 days were used for all experiments. Neurons were plated in 25 cm^2^ cell culture flasks with a vented cap and treated with 150 μM KA and the indicated PF‐4708671. Cultures were lysed in RIPA buffer and analyzed by SDS–PAGE followed by Western blotting as described above. For immunostaining, neurons were plated in 48‐well plates, and 20 μM PF‐4708671 was added to the cells 6 h before the addition of KA. Plates were immunostained using an mAb against β‐tubulin(III) (1:2000, chicken, ab41489; Abcam) and imaged under an inverted fluorescence microscope (Eclipse Ti‐U, Nikon) (see Supporting Information).

### 2.11 Golgi‐Cox staining

As described previously,^26^ Golgi staining was performed following the manufacturer's protocols (Hito Golgi‐Cox OptimStain™ Kit). A total of 22–25 dendrites of pyramidal neurons located in the hippocampal CA1 region per group (*N* = 4–5) were imaged under bright‐field illumination (T‐PMT) on a Zeiss microscope system (LSM 780) with a 100×/1.4 oil immersion lens and evaluated by FIJI software (Fiji, ImageJ 1.46, NIH).

### 2.12 Statistical analysis

Statistical tests were performed using Prism GraphPad 8.0. The Shapiro–Wilk or Kolmogorov–Smirnov test was used to assess normality, and the Brown‐Forsythe or *F* test was used to assess homoscedasticity. The histogram lay within normal distributions and the variance between groups was homogeneous. Statistical significance was determined by comparing the means of different groups using *unpaired Student's t‐test*, *one‐way* or *two‐way* ANOVA and post hoc analysis. Welch's ANOVA test was used for the statistical significance of normally distributed with heterogeneous variance data. The Kruskal–Wallis test was used for the statistical significance of non‐normally distributed data. In all figures, data are represented as the means ± SEMs. A *p* value of <0.05 was considered statistically significant.

## 3 RESULTS

### 3.1 The highly selective S6K1 inhibitor PF‐4708671 protected neurites from KA‐induced excitotoxicity

**FIGURE 1:**
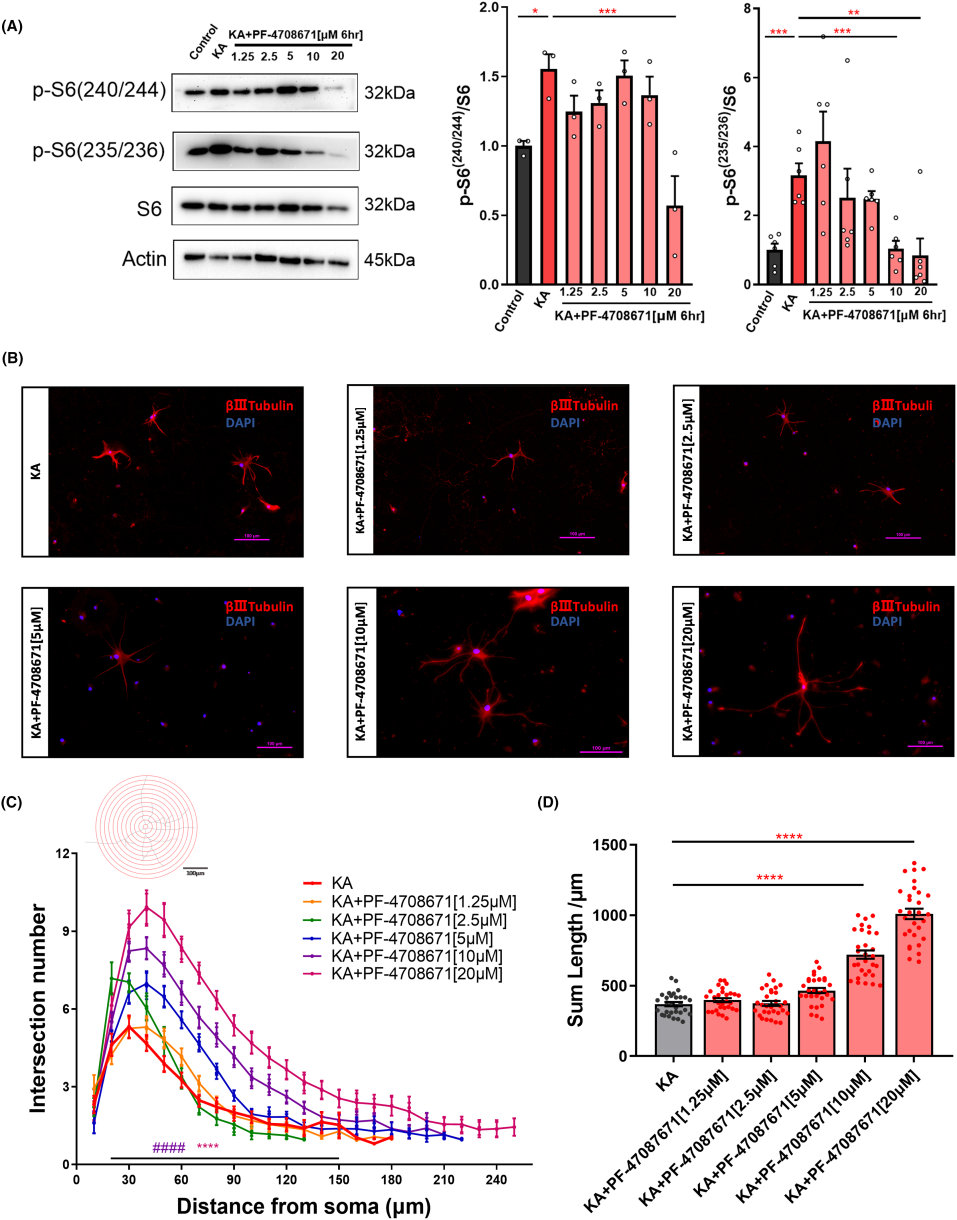
The highly selective S6K1 inhibitor PF‐4708671 protected neurites from KA‐induced excitotoxicity. (A) Phosphorylation level of S6 in primary hippocampal neurons following treatment with KA and PF‐4708671 at the indicated concentrations. Whole hippocampal cell lysates were used for Western blot analysis. All the ratios shown in the cumulative graph were normalized first to the levels of total S6 and then expressed relative to the control (DMSO). Data are represented as the mean ± SEM (*n* = 3–6 replicates per condition from a single preparation of primary neurons, *one‐way* ANOVA, Tukey's posttest, *p < 0.05, **p < 0.01 and ***p < 0.001). (B) Primary hippocampal neurons treated with the indicated PF‐4708671 for 6 h before KA treatment. Immunofluorescence (IF) detection of neuron morphology by neuronal marker βIII‐tubulin (Red), Blue represents nuclear stain. Scale bar, 100 μm. (C and D) PF‐4708671 increases (C) total Sholl intersections and (D) total process length in primary hippocampal neurons following treatment with KA (at least 20 neurons from four to six replicates per condition from a single preparation of primary neurons, (C) Welch's ANOVA test and (D) *one‐way* analysis of variance (ANOVA) with Bonferroni's multiple comparisons test). Data are shown as the mean ± SEM. (****p < 0.0001, ^####^ *p* < 0.0001).

KA‐induced excitotoxicity has been validated to induce dendrite injury in hippocampal neurons in vitro.^24^ To assess the effect of S6K1 inhibition on KA‐induced excitotoxicity axonal outgrowth in vitro, we treated primary hippocampal neurons with 150 μM KA and the indicated concentrations of PF‐4708671 at DIV 12. We carried out a dose‐dependent Western blot analysis in whole hippocampal lysates. We examined the phosphorylation state of key translational control molecules regulated by S6K1 in KA‐induced excitotoxicity. Compared with the control (dimethyl sulfoxide [DMSO]), the levels of pS6 (240/44 and 235/36) were significantly increased in KA‐treated neurons and reversed by 20 and 10 μM PF‐4708671, respectively (Figure 1A). Morphological studies showed that PF‐4708671 induced a dose‐dependent increase in neurite outgrowth relative to KA (DMSO) [369.4 ± 14.41], reaching ~200% at 10 μM (721.1 ± 29.42) (Figure 1B,D). In addition, we measured the alterations in neurite complexity by concentric circle (Sholl's) analysis and found that neurite arborization (Figure 1C) significantly increased in the 10 μM group compared with the KA (DMSO) group. Moreover, the decrease in 235/36 phosphorylation was correlated with the induction of neurite outgrowth (Figure S1). These data strongly suggest that S6K1/rpS6 can regulate neuronal growth/development through a mechanism that is dependent on translation in KA‐induced excitotoxicity.

### 3.2 The effects of PF‐4708671 on seizure episodes recorded in the CA1 region of the hippocampus in chronic epileptic rats

**FIGURE 2:**
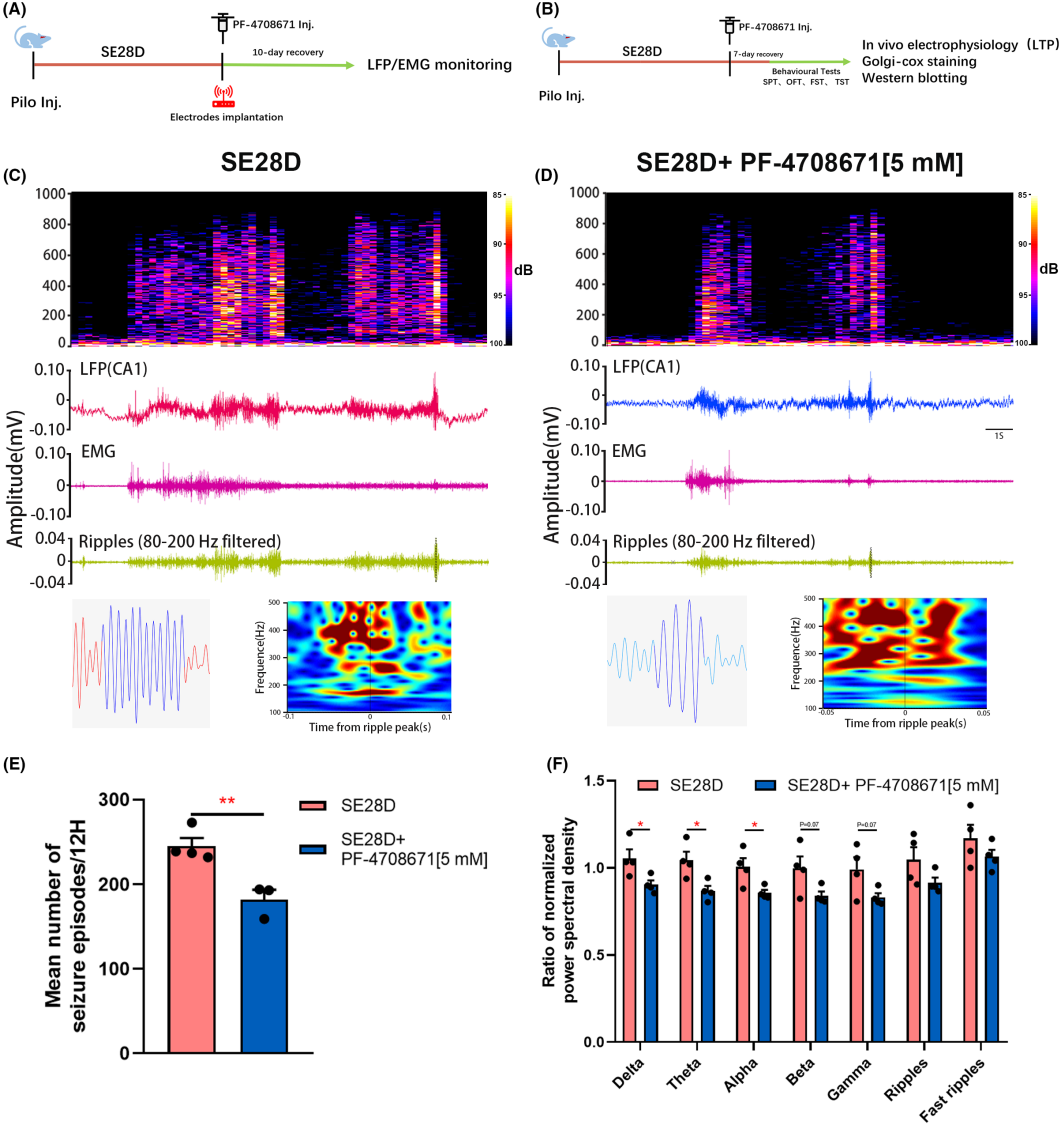
The effects of PF‐4708671 on seizure episodes recorded in the CA1 region of the hippocampus in chronic epileptic rats. (A and B) Schematic diagram of the experimental design. (A) Experimental timeline of the electrode implantation and LFP/EMG monitoring. (B) Experimental timeline of the behavioral Tests, in vivo electrophysiology, Golgi‐Cox staining, and western blotting. (C and D) Total power of the seizure episodes of local field potential (LFP) (top) recorded in the CA1 derived from the SE28D (A) and SE28D+PF‐4708671[5 mM] (B) group, as well as the representative seizure episodes for LFP, electromyogram (EMG) and filtered ripple events (middle), and a representative ripple (left panel) and spectrogram (right panel) from filtered ripples events (bottom). (E) Mean number of seizure episodes. Data are plotted as the mean ± SEM (*n* = 3–4), **p < 0.01 by *unpaired t test*. (F) Comparison of the normalized power spectral intensity of the LFP frequency spectrum of seizure episodes in CA1. Data are plotted as the mean ± SEM (*n* = 4), *p < 0.05 by *unpaired t test*.

Considering that S6K1 can regulate neuron growth/development in vitro, we investigated whether S6K1 has a regulatory effect on seizure episodes in the CA1 region of the rat hippocampus. Our results showed that the total number of seizure episodes recorded in the CA1 region was significantly reduced following treatment with 5 mM PF‐4708671 compared with the SE28D group (*p* < 0.01, Figure 2E). In addition, the ratio of PSD in the δ, θ and α bands was significantly reduced in the SE28D+5 mM PF‐4708671 group compared with the SE28 group (*p* < 0.05, Figure 2F). There was also a trend of reduction in the β and γ bands. In addition, there was no significant difference in the ratio of PSD in the ripple and fast ripple bands between the two groups. These results indicate that inhibiting S6K1 attenuates behavioral seizures and restores abnormal power changes.

### 3.3 PF‐4708671 modulates depression but not anxiety‐like behavior in chronic epileptic rats

**FIGURE 3:**
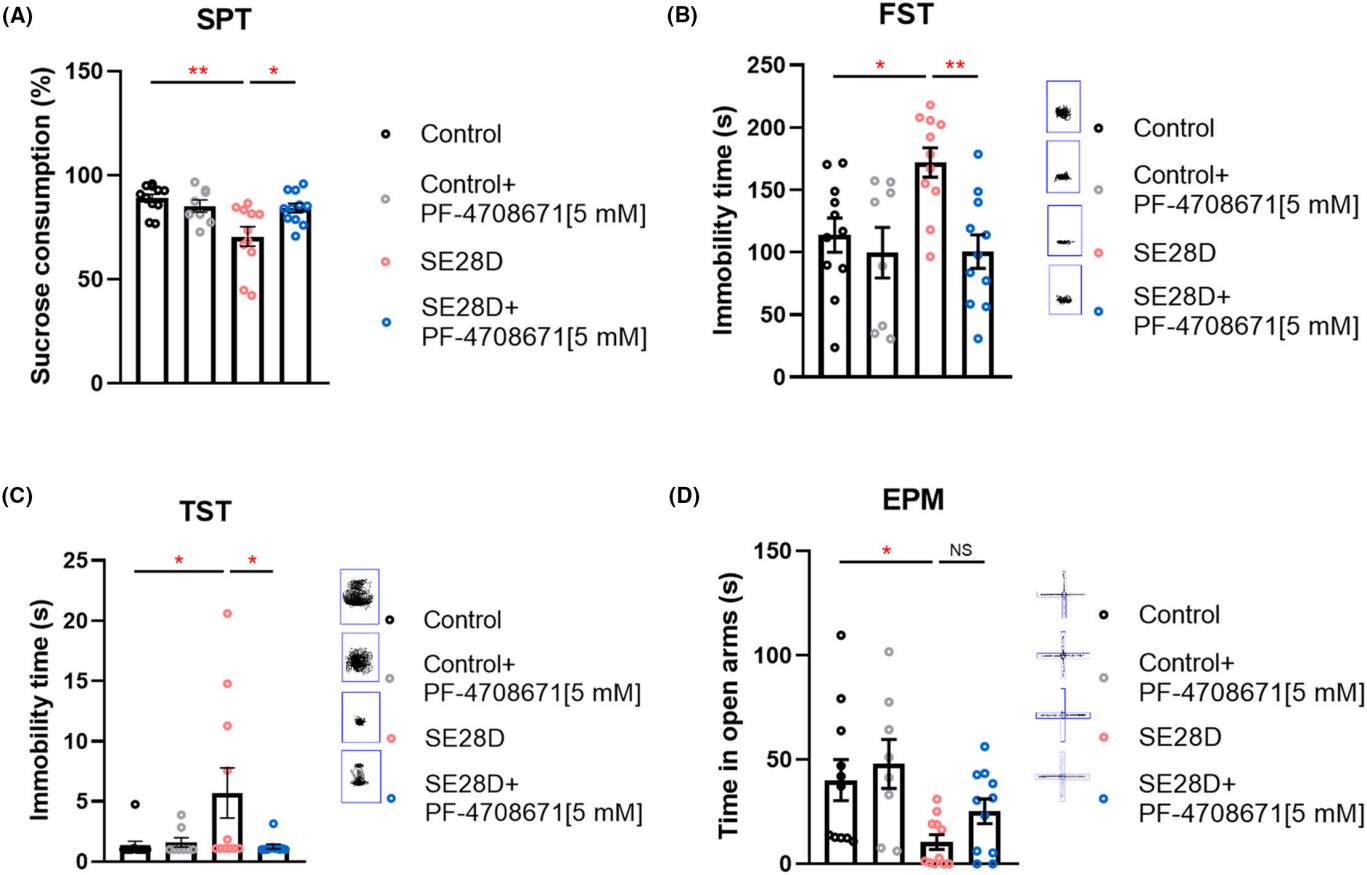
PF‐4708671 modulates depression but not anxiety‐like behavior in chronic epileptic rats. (A–C) Depression‐like behaviors of the rats were evaluated using (A) percentage sucrose consumption in the sucrose preference test (SPT), (B) immobility time in the forced swimming test (FST), and (C) immobility time in the tail suspension test (TST). (DMSO Control: *n* = 11, Control+PF‐4708671[5 mM]: *n* = 8, SE28D: *n* = 11, SE28D+PF‐4708671[5 mM]: *n* = 11) Data are plotted as the mean ± SEM and were analyzed by *one‐way* ANOVA with the Tukey's multiple comparisons test or Kruskal–Wallis test with Dunn's multiple comparisons test, *p < 0.05, **p < 0.01. (D) Anxiety‐like behaviors of the rats were evaluated using time spent in the open arms of the elevated plus maze (EPM). (DMSO Control: *n* = 11, Control+PF‐4708671[5 mM]: *n* = 8, SE28D: *n* = 11, SE28D+PF‐4708671[5 mM]: *n* = 11) Data are plotted as the mean ± SEM and were analyzed by *one‐way* ANOVA with Tukey's multiple comparisons test, *p < 0.05.

Patients with epilepsy have a high‐comorbidity burden. Multiple conditions and diseases, including depression, anxiety, and dementia, are up to eight times more common in people with epilepsy than in the general population.^3^ We performed a variety of behavioral tests to assess depression‐ and anxiety‐like behaviors in chronic epileptic rats. We first assessed the presence of anhedonia, a hallmark symptom of depression, in chronic epileptic rats using the SPT. We next tested depression‐like behavior using the FST and TST, where immobility was used as a measure of behavioral hopelessness. We found that under control conditions, chronic epileptic rats showed no sucrose preference and were immobile for a greater duration than control rats, consistent with a depressive phenotype. Inhibition of S6K1 using PF‐4708671 injection successfully corrected many behavioral abnormalities (Figure 3A–C). In the EPM, a test of anxiety‐like behavior, there was an anxiogenic effect of seizures, but there was no significant difference between the epilepsy rat and the S6K1‐inhibited rat (Figure 3D). Taken together, our behavioral results suggest that inhibiting S6K1 reduces behavioral disorder complications such as anhedonia or “behavioral despair”.

### 3.4 PF‐4708671 reverses CA3–CA1 long‐term potentiation (LTP) deficits in vivo

Various animal models of epilepsy display significantly attenuated hippocampal LTP in vitro.^27^ Previous studies have shown that the mTORC1‐S6K1 axis regulates fundamental cellular processes, including transcription, translation, and protein synthesis.^28^ We reasoned that if S6K1 plays a role in gene expression, characterization of its LTP would yield insights into the potential impact of synaptic local translation on epilepsy synaptic plasticity. We induced CA3–CA1 LTP in vivo in four groups.

**FIGURE 4:**
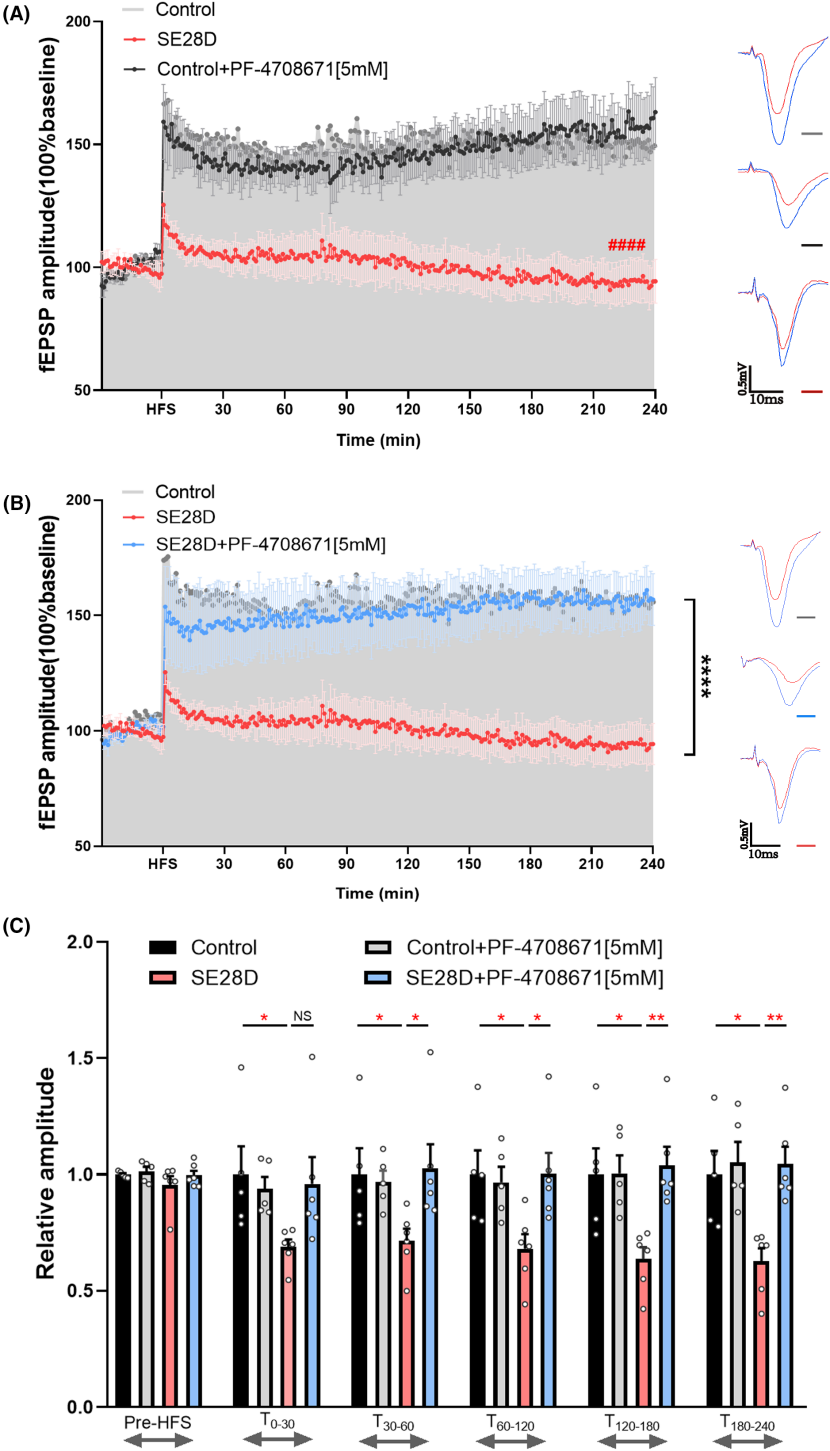
PF‐4708671 reverses CA3–CA1 long‐term potentiation (LTP) deficits in vivo. (A and B) High‐frequency stimulation in the CA3 area of the hippocampus induces LTP in the CA1 region. (A) In vivo, chronic epileptic rats displayed a deficit in HFS‐induced CA3–CA1 LTP compared with control rats, and (B) PF‐4708671 reversed this deficit. Left, summary plots of normalized fEPSPs in anesthetized rats under DMSO control (*n* = 5), control+PF‐4708671[5 mM] (*n* = 5), SE28D (*n* = 6) and SE28D+PF‐4708671[5 mM] (*n* = 6). Dots are the mean ± SEM of the normalized fEPSP amplitude for 1 min periods. *Two‐way* ANOVA with repeated measures (Groups × Time) showed significant differences between control vs. SE28D (^####^ *p* < 0.0001) or SE28D vs. SE28D+PF‐4708671[5 mM] (****p < 0.0001). Right, Representative fEPSPs were recorded from the CA1 region of each group of animals before (red) and after (blue) hippocampal HFS. (C) Bar histograms of normalized fEPSPs from the experiment in (A and B) 240 min after HFS. Data, mean ± SEM, *one‐way* ANOVA with Dunnett's multiple comparisons test. *p < 0.05, **p < 0.01. HFS, high frequency stimulation.

As shown in Figure 4 and Figures S2,S3, an immediate and prolonged increase in the amplitude of CA1 potentials was observed immediately after the conditioning stimuli in DMSO controls (166.9% ± 16.78% [*n* = 5], *p* < 0.005 vs. baseline). Consistent with previous in vitro findings, we observed attenuated LTP in chronic epileptic rats, and LTP was significantly attenuated in chronic epileptic rats (93.79% ± 0.2189% [*n* = 6], *p* < 0.0001 vs. Control) (Figure 4A). No significant difference was found in the LTP amplitude between the control (sham) and control (DMSO) (*p* = 0.9924) and the DMSO control and control+PF‐4708671 groups (*p* = 0.2550) (Figure S3 and Figure 4A).

The LTP recovered significantly and slowly after treatment with 5 mM PF‐4708671 in the CA1 of chronic epileptic rats (156.1% ± 0.3765% [*n* = 6], *p* < 0.0001 vs. SE28D, *t*_0–30 min_, 1.027 ± 0.1037 vs. 0.7156 ± 0.05186, *p* = 0.1026; *t*_30–60 min_, 0.9596 ± 0.1149 vs. 0.6886 ± 0.03204, *p* < 0.05; *t*_60–120 min_, 1.004 ± 0.08888 vs. 0.6814 ± 0.06208, *p* < 0.05; *t*_120–180 min_, 1.040 ± 0.07961 vs. 0.6363 ± 0.05114, *p* < 0.01; *t*_180–240 min_, 1.045 ± 0.07398 vs. 0.6263 ± 0.05633, *p* < 0.01) (Figure 4B,C).

### 3.5 PF‐4708671 alleviates spine loss and dendritic pathology in the CA1 region of the hippocampus in chronic epileptic rats

**FIGURE 5:**
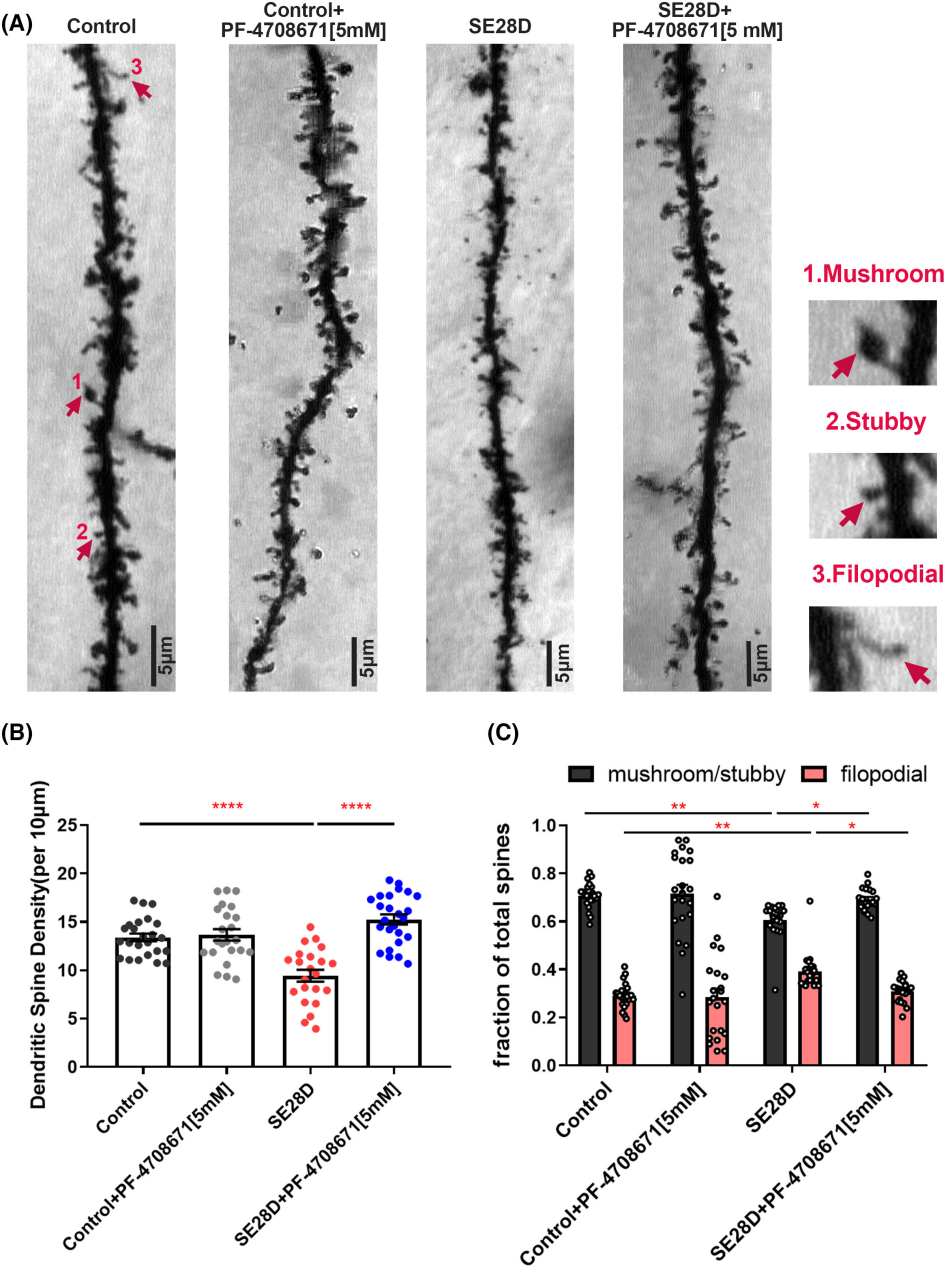
PF‐4708671 alleviates spine loss and dendritic pathology in the CA1 region of the hippocampus in chronic epileptic rats. (A) Representative images of Golgi‐Cox‐stained CA1 secondary branches of apical dendrites from all four groups, original magnification 100× Oil. Scale bar = 5 μm. Representative images of mushroom, stubby and filopodial (left). (B) Relative quantification of dendritic spines in CA1: Number of spines/10 μm: *n* = 22–25 dendrites (four or five neurons per rat, five rats), Scale bar = 10 μm. Data are plotted as the mean ± SEM, ****p < 0.0001 by *one‐way* ANOVA with Tukey's multiple comparisons test. (C) Spine Morphology Study shows fractional filopodial and stubby/mushroom spines in CA1: Number of spines/10 μm: *n* = 20–23 dendrites (four or five neurons per rat, five rats), Scale bar = 10 μm. Data are plotted as the mean ± SEM, *p < 0.05, **p < 0.01 by *two‐way* ANOVA with Tukey's multiple comparisons test.

Abnormal dendritic morphology and spine loss have been reported both in animal models of epilepsy and human brain tissue from epilepsy patients.^29^ Our preliminary studies found that PF4708671 effectively protects against KA‐induced neurite damage in primary hippocampal neurons and reverses in vivo CA3–CA1 LTP deficits in epileptic rats. To verify structural plasticity, we measured spine density and morphology in the hippocampal CA1 region using Golgi‐cox staining. We found that the spine generation and fraction of stubby and mushroom spines significantly decreased in the chronic epileptic rats compared with the control, whereas treatment with 5 mM PF‐4708671 in hippocampal CA1 by stereotactic injection abolished these changes (Figure 5A–C). Spine classification analysis also revealed a lower fraction of mature spines (mushroom spines) compared to stubby, filopodia‐like, and thin spines viewed as immature structures in chronic epileptic rats (Figure S4). The integrated results of synaptic function and structure suggest that S6K1 negatively regulates postsynaptic plasticity of the CA3–CA1 circuit in chronic epileptic rats.

### 3.6 PF‐4708671 regulates the expression levels of synaptic plasticity proteins involved in LTP induction and maintenance in chronic epileptic rats

**FIGURE 6:**
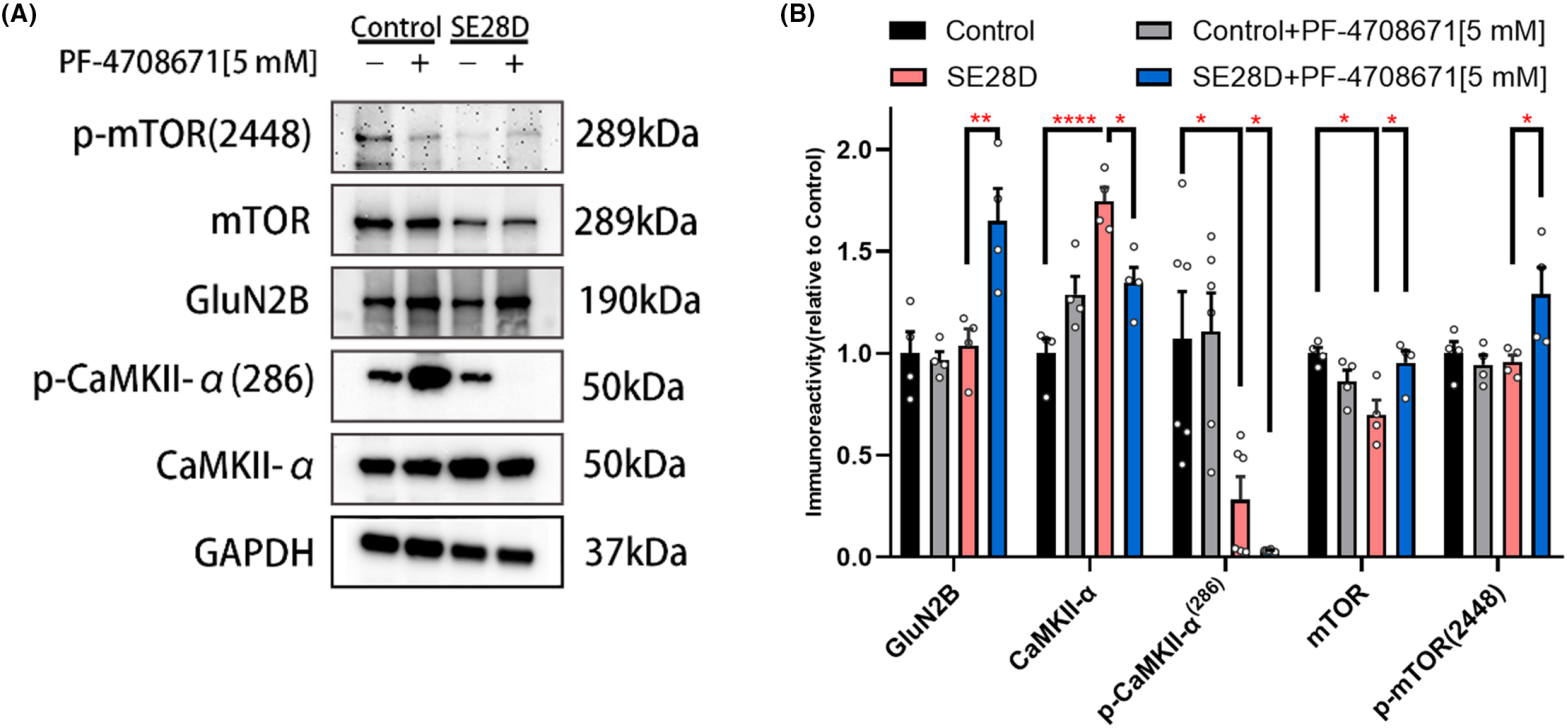
PF‐4708671 regulates the expression levels of synaptic plasticity proteins involved in LTP induction and maintenance in chronic epileptic rats. (A) Representative Western blot images of synaptic plasticity proteins in synaptosomes in CA1. (B) Quantification of synaptic plasticity proteins in synaptosomes with GAPDH as a control for protein loading (DMSO Control: *n* = 4–6, Control+PF‐4708671[5 mM]: *n* = 4–6, SE28D: *n* = 4–6, SE28D+PF‐4708671[5 mM]: *n* = 4–6). Data were plotted as the mean ± SEM and were analyzed by *one‐way* ANOVA with Tukey's multiple comparisons test, *p < 0.05, **p < 0.01, ****p < 0.0001.

To further explore how S6K1 affects synaptic plasticity by regulating the translation of synaptic proteins, we detected the expression levels of synaptic proteins involved in the regulation of LTP induction and maintenance, including calcium/calmodulin‐dependent protein kinase IIα (CaMKIIα), GluN2B, and phospho‐CaMKII (Thr286) (Figure 6A,B). In parallel, we also examined the levels of phospho‐mTOR (S2448) and mTOR, which are regulated by the negative feedback of S6K1^30^ (Figure 6A). We observed a remarkable increase in the level of CaMKIIα (*p* < 0.0001 vs. Control) and a decrease in mTOR (*p* < 0.05 vs. Control) in chronic epileptic rats, and both could be reversed by PF‐4708671(Figure 6B). Interestingly, the application of PF‐4708671 further decreased the expression of phospho‐CaMKII (Thr286) (*p* < 0.05 vs. SE28D) while increasing the expression of GluN2B (*p* < 0.01 vs. SE28D) in chronic epileptic rats (Figure 6B). In summary, these findings suggest that S6K1 is involved in balancing the expression of synapse‐associated proteins.

## 4 DISCUSSION

In this study, we pharmacologically inhibited S6K1, a translational control molecule, for the first time in epileptic rats. Efforts were made to link the molecular and biochemical changes associated with S6K1 activity to changes in synaptic function to correct the phenotype shown in epileptic rats. We found that decreased phosphorylation of the substrate of S6K1, S6, strongly promotes neurite outgrowth and spine maturation. The association of S6K1 with synaptic function in the hippocampus of epileptic rats was further validated by electrophysiology, providing strong evidence that S6K1 is involved in the regulation of synaptic local structure and function to correct epileptic seizures and depression‐like behavior in epileptic rats.

Previous studies have shown that S6K1 is a negative regulator of neurite growth in primary neurons.^17^ Consistent with this study, inhibition of S6K1 protected and promoted neuronal growth in KA‐treated cultured neurons. In light of this result, we investigated for the first time the effect of S6K1 inhibition on behavioral seizures and psychiatric comorbidities in vivo. Rhythmic oscillations in the neuronal system can maintain normal physiological function or may interfere with normal neurological function. Recurrent seizures have been shown to interfere with rhythmic oscillations, and attempts are being made to predict and treat epilepsy by looking for pathological oscillations.^31^ We evaluated various oscillations and found that inhibition of S6K1 decreased the activity of low‐frequency (1–80 Hz) oscillations. Delta and theta oscillations are abnormally increased in epilepsy, and it is thought that abnormal increases in theta oscillations can be considered a “forerunner” to seizures.^32^, ^33^ This provides intuitive neural network signaling evidence that inhibition of S6K1 reduces seizure behavior. The fluctuations in oscillations also reflect neural network‐level signals that mediate depression/anxiety‐like behavior.^34^ Increasing research has demonstrated that inhibition of S6K1 improves a variety of socioemotional responses and learning and memory behavior disorders.^10^, ^14^ In line with previous findings, we observed increased depression/anxiety‐like behavior in epileptic rats.^35^, ^36^ S6K1 is somewhat effective in bringing back harmonious oscillatory activity in the brain, thereby reducing depression‐like behavior. This is also the first direct visualization of how S6K1 modulates neural activity mediating behavioral changes in neurological disorders. Unexpectedly, our study shows that pharmacological inhibition of S6K1 neither reduced nor increased anxiety‐like behavior in rats. Previous studies have shown that genetic removal of S6K1 in mice increases anxiety‐like behavior,^37^ and these results provide a basis for understanding reports of anxiety following mTOR inhibitor treatment.^38^ It seems that injection of small molecule inhibitors is preferable to direct manipulation of gene expression in the context of cooccurring anxiety in epilepsy.

Moreover, theta oscillations are usually associated with synaptic plasticity and behavioral memory.^39^ This further supports the idea that S6K1 is involved in the regulation of synaptic plasticity.

Neural oscillations are thought to provide a temporal structure that precisely regulates synaptic plasticity and thus determines whether the strengthening or weakening of synaptic connections occurs.^40^ Therefore, we investigated how S6K1 regulates synaptic plasticity in response to altered neural activity. Consistent with reports of hippocampal LTP attenuation, altered dendritic pathology, and spinal loss in hippocampal neurons in human brain tissue from patients with epilepsy or animal models of epilepsy,^27^, ^29^ we found that inhibition of S6K1 counteracted the abnormal dendritic morphology due to recurrent seizures. Interestingly, the application of PF‐4708671 did not aggravate but rather improved the impairment of potentiation, consistent with previous studies.^14^ Indeed, recovery of LTP was a slow process. Inhibiting S6K1 enhanced LTP maintenance but did not completely reverse the early stages of induction. Previous studies have suggested that the induction phase of protein synthesis‐dependent LTP is sensitive to rapamycin and that dendritic p70S6K activation is necessary for the induction of late long‐term potentiation (L‐LTP) at Schaffer collateral commissural fiber‐CA1 synapses.^41^ This is also demonstrated by our data, which does not contradict the role of synaptic protein synthesis in plasticity. The most parsimonious explanation is that S6K1 does not directly affect the synthesis of proteins at synapses but rather regulates the functional state of synapses, just as the synaptic tag theory does, which suggests that LTP initiates the creation of short‐duration “synaptic tags” unrelated to protein synthesis at enhanced synapses.^42^

In the study of LTP, the molecular mechanisms involved in L‐LTP induction are thought to be similar to those of E‐LTP and are associated with the activation of multiple protein kinases.^43^ CaMKII plays an important role in synaptic plasticity. Previous studies have shown that S6K1 is involved in the regulation of CaMKII.^10^ To further explore how S6K1 affects LTP by regulating CaMKII, we examined related synaptic plasticity proteins and found that S6K1 inhibits CaMKII phosphorylation at T286 to a large extent and increases GluN2B expression. CaMKII and its autophosphorylation at T286 are required for LTP induction.^44^ It is unclear whether LTP maintenance requires T286 phosphorylation. Some studies suggest that phosphorylation of T286 only acts in the early stages of LTP induction, whereas there are studies supporting that T286 phosphorylation is essential during LTP maintenance.^45^ The electrophysiological and protein blotting data suggest that autophosphorylation at T286 is not required for the maintenance phase of L‐LTP. These findings support the notion that the maintenance phase of L‐LTP is preserved in S6K1‐inhibited epileptic rats via CaMKII‐GluN2B binding rather than T286 phosphorylation, which helps to understand the potential mechanism by which S6K1/2‐deficient mice do not exhibit alterations in L‐LTP and early‐onset contextual fear memory deficits.^46^

Numerous studies support the idea that S6K1 participates in regulatory feedback mTOR signaling.^10^ In addition, our results showed an increased level of mTOR phosphorylation at Ser‐2448, which overlaps with reports from previous studies that phosphorylation of mTOR at Ser‐2448 was mediated by S6K1.^47^ This allows inhibition of S6K1 to simultaneously regulate normal protein synthesis by activating the mTOR/4E‐BP (eIF4E‐binding protein) pathway, which explains the potential mechanism by which deficient LTP can be recovered in epileptic rats.

In summary, we elucidated for the first time that S6K1 has antiepileptic and antidepressive effects in epilepsy and successfully restores low‐frequency power in epileptic rats, providing more evidence for its potential role in the central nervous system. In particular, ongoing phase III clinical trials of S6K1 inhibitors in non‐CNS diseases make them an encouraging potential therapeutic target for CNS diseases, especially when the clinical availability of mTOR inhibitors is limited by a multitude of side effects.^48^, ^49^

## AUTHOR CONTRIBUTIONS

Conceptualization, W.L., H.H., and C.Z.; methodology, Y.Z. (Yuying Zhang) and L.W.; software, Y.Z. (Yuying Zhang); formal analysis, Y.Z. (Yuying Zhang) and X.C.; investigation, Y.Z. (Yuying Zhang); resources, W.L. and H.H.; data curation, Y.Z. (Yuying Zhang); writing—original draft preparation, Y.Z. (Yuying Zhang); writing—review and editing, Y.Z. (Yuying Zhang) and C.L.; visualization, J.L. and M.W.; supervision, Y.Z. (Yuying Zhang); project administration, Y.Z. (Yuying Zhang) and W.L.; funding acquisition, W.L. and H.H. All the authors have read and agreed to the published version of the manuscript.

## FUNDING INFORMATION

This work was supported by grants from National Natural Science Foundation of China (No. 82171445); The Youth Project of National Natural Science Foundation of China (No. 81901311).

## CONFLICT OF INTEREST STATEMENT

The authors have no actual or potential conflicts of interest to declare. We confirm that we have read the Journal's position on issues involved in ethical publication and affirm that this report is consistent with those guidelines.

## Supporting information


Appendix S1.

## Data Availability

The data used for this study, though not available in a public repository, will be made available from the corresponding author upon reasonable request.

## References

[1] Thijs RD , Surges R , O'Brien TJ , Sander JW . Epilepsy in adults. Lancet. 2019;393(10172):689‐701. doi:10.1016/S0140-6736(18)32596-0 30686584

[2] Krishnan V . Depression and anxiety in the epilepsies: from bench to bedside. Curr Neurol Neurosci Rep. 2020;20(9):41. doi:10.1007/s11910-020-01065-z 32666148 PMC9157472

[3] Keezer MR , Sisodiya SM , Sander JW . Comorbidities of epilepsy: current concepts and future perspectives. Lancet Neurol. 2016;15(1):106‐115. doi:10.1016/S1474-4422(15)00225-2 26549780

[4] Patel DC , Tewari BP , Chaunsali L , Sontheimer H . Neuron‐glia interactions in the pathophysiology of epilepsy. Nat Rev Neurosci. 2019;20(5):282‐297. doi:10.1038/s41583-019-0126-4 30792501 PMC8558781

[5] Hauser RM , Henshall DC , Lubin FD . The epigenetics of epilepsy and its progression. Neuroscientist. 2018;24(2):186‐200. doi:10.1177/1073858417705840 28468530

[6] Rajgor D , Welle TM , Smith KR . The coordination of local translation, membranous organelle trafficking, and synaptic plasticity in neurons. Front Cell Dev Biol. 2021;9:711446. doi:10.3389/fcell.2021.711446 34336865 PMC8317219

[7] Malone TJ , Kaczmarek LK . The role of altered translation in intellectual disability and epilepsy. Prog Neurobiol. 2022;213:102267. doi:10.1016/j.pneurobio.2022.102267 35364140 PMC10583652

[8] Switon K , Kotulska K , Janusz‐Kaminska A , Zmorzynska J , Jaworski J . Molecular neurobiology of mTOR. Neuroscience. 2017;341:112‐153. doi:10.1016/j.neuroscience.2016.11.017 27889578

[9] Dorrello NV , Peschiaroli A , Guardavaccaro D , Colburn NH , Sherman NE , Pagano M . S6K1‐ and betaTRCP‐mediated degradation of PDCD4 promotes protein translation and cell growth. Science. 2006;314(5798):467‐471. doi:10.1126/science.1130276 17053147

[10] Bhattacharya A , Kaphzan H , Alvarez‐Dieppa AC , Murphy JP , Pierre P , Klann E . Genetic removal of p70 S6 kinase 1 corrects molecular, synaptic, and behavioral phenotypes in fragile X syndrome mice. Neuron. 2012;76(2):325‐337. doi:10.1016/j.neuron.2012.07.022 23083736 PMC3479445

[11] Bonucci M , Kuperwasser N , Barbe S , et al. mTOR and S6K1 drive polycystic kidney by the control of Afadin‐dependent oriented cell division. Nat Commun. 2020;11(1):3200. doi:10.1038/s41467-020-16978-z 32581239 PMC7314806

[12] Pelorosso C , Watrin F , Conti V , et al. Somatic double‐hit in MTOR and RPS6 in hemimegalencephaly with intractable epilepsy. Hum Mol Genet. 2019;28(22):3755‐3765. doi:10.1093/hmg/ddz194 31411685 PMC6935386

[13] Gu J , Tian X , Wang W , et al. Inhibition of Cgkii suppresses seizure activity and hippocampal excitation by regulating the postsynaptic delivery of Glua1. Cell Physiol Biochem. 2018;46(1):160‐177. doi:10.1159/000488419 29587280

[14] Sun J , Liu Y , Tran J , O'Neal P , Baudry M , Bi X . mTORC1‐S6K1 inhibition or mTORC2 activation improves hippocampal synaptic plasticity and learning in Angelman syndrome mice. Cell Mol Life Sci. 2016;73(22):4303‐4314. doi:10.1007/s00018-016-2269-z 27173058 PMC5056144

[15] Zhu C , Lin R , Liu C , et al. The antagonism of 5‐HT6 receptor attenuates current‐induced spikes and improves long‐term potentiation via the regulation of M‐currents in a pilocarpine‐induced epilepsy model. Front Pharmacol. 2020;11:475. doi:10.3389/fphar.2020.00475 32425770 PMC7212420

[16] Racine RJ . Modification of seizure activity by electrical stimulation. II Motor seizure. Electroencephalogr Clin Neurophysiol. 1972;32(3):281‐294. doi:10.1016/0013-4694(72)90177-0 4110397

[17] Al‐Ali H , Ding Y , Slepak T , Wu W , Sun Y , Martinez Y . The mTOR substrate S6 kinase 1 (S6K1) is a negative regulator of axon regeneration and a potential drug target for central nervous system injury. J Neurosci. 2017;37(30):7079‐7095. doi:10.1523/JNEUROSCI.0931-17.2017 28626016 PMC5546395

[18] Bagur S , Lacroix MM , de Lavilléon G , Lefort JM , Geoffroy H , Benchenane K . Harnessing olfactory bulb oscillations to perform fully brain‐based sleep‐scoring and real‐time monitoring of anaesthesia depth. PLoS Biol. 2018;16(11):e2005458. doi:10.1371/journal.pbio.2005458 30408025 PMC6224033

[19] Li D , Luo D , Wang J , et al. Electrical stimulation of the endopiriform nucleus attenuates epilepsy in rats by network modulation. Ann Clin Transl Neurol. 2020;7(12):2356‐2369. doi:10.1002/acn3.51214 33128504 PMC7732253

[20] Guan J , Ding Y , Rong Y , et al. Early life stress increases brain glutamate and induces neurobehavioral manifestations in rats. ACS Chem Nerosci. 2020;11(24):4169‐4178. doi:10.1021/acschemneuro.0c00454 33179901

[21] Li J , Burgess DJ . Biomarker monitoring and long‐acting insulin treatment in a stress model to facilitate personalized diabetic control. J Control Release. 2021;332:21‐28. doi:10.1016/j.jconrel.2021.02.013 33600878

[22] Detke MJ , Rickels M , Lucki I . Active behaviors in the rat forced swimming test differentially produced by serotonergic and noradrenergic antidepressants. Psychopharmacology (Berl). 1995;121(1):66‐72. doi:10.1007/BF02245592 8539342

[23] Parihar VK , Hattiangady B , Kuruba R , Shuai B , Shetty AK . Predictable chronic mild stress improves mood, hippocampal neurogenesis and memory. Mol Psychiatry. 2011;16(2):171‐183. doi:10.1038/mp.2009.130 20010892 PMC2891880

[24] Xiang Y , Niu Y , Xie Y , et al. Inhibition of RhoA/rho kinase signaling pathway by fasudil protects against kainic acid‐induced neurite injury. Brain Behav. 2021;11(8):e2266. doi:10.1002/brb3.2266 34156163 PMC8413774

[25] Mattson MP , Dou P , Kater SB . Outgrowth‐regulating actions of glutamate in isolated hippocampal pyramidal neurons. J Neurosci. 1988;8(6):2087‐2100. 2898515 10.1523/JNEUROSCI.08-06-02087.1988 PMC6569320

[26] Cheng X , Wu X , Zhang Y , et al. LRRK2 deficiency aggravates sleep deprivation‐induced cognitive loss by perturbing synaptic pruning in mice. Brain Sci. 2022;12(9):1‐13. doi:10.3390/brainsci12091200 PMC9496729 36138936

[27] Sorensen AT , Nikitidou L , Ledri M , et al. Hippocampal NPY gene transfer attenuates seizures without affecting epilepsy‐induced impairment of LTP. Exp Neurol. 2009;215(2):328‐333. doi:10.1016/j.expneurol.2008.10.015 19038255 PMC2896682

[28] Magnuson B , Ekim B , Fingar DC . Regulation and function of ribosomal protein S6 kinase (S6K) within mTOR signalling networks. Biochem J. 2012;441(1):1‐21. doi:10.1042/BJ20110892 22168436

[29] Rossini L , de Santis D , Mauceri RR , et al. Dendritic pathology, spine loss and synaptic reorganization in human cortex from epilepsy patients. Brain. 2021;144(1):251‐265. doi:10.1093/brain/awaa387 33221837

[30] Shimobayashi M , Hall MN . Making new contacts: the mTOR network in metabolism and signalling crosstalk. Nat Rev Mol Cell Biol. 2014;15(3):155‐162. doi:10.1038/nrm3757 24556838

[31] Foldi T , Lorincz ML , Berenyi A . Temporally targeted interactions with pathologic oscillations as therapeutical targets in epilepsy and beyond. Front Neural Circuits. 2021;15:784085. doi:10.3389/fncir.2021.784085 34955760 PMC8693222

[32] Vrinda M , Sasidharan A , Aparna S , Srikumar BN , Kutty BM , Shankaranarayana Rao BS . Enriched environment attenuates behavioral seizures and depression in chronic temporal lobe epilepsy. Epilepsia. 2017;58(7):1148‐1158. doi:10.1111/epi.13767 28480502

[33] Pasquetti MV , Meier L , Marafiga JR , Barbieri Caus L , Tort ABL , Calcagnotto ME . Hippocampal CA1 and cortical interictal oscillations in the pilocarpine model of epilepsy. Brain Res. 2019;1722:146351. doi:10.1016/j.brainres.2019.146351 31351038

[34] Hultman R , Ulrich K , Sachs BD , et al. Brain‐wide electrical spatiotemporal dynamics encode depression vulnerability. Cell. 2018;173(1):166‐180 e14. doi:10.1016/j.cell.2018.02.012 29502969 PMC6005365

[35] Shen Y , Peng W , Chen Q , et al. Anti‐inflammatory treatment with a soluble epoxide hydrolase inhibitor attenuates seizures and epilepsy‐associated depression in the LiCl‐pilocarpine post‐status epilepticus rat model. Brain Behav Immun. 2019;81:535‐544. doi:10.1016/j.bbi.2019.07.014 31306773 PMC6873816

[36] Faure JB , Marques‐Carneiro JE , Akimana G , et al. Attention and executive functions in a rat model of chronic epilepsy. Epilepsia. 2014;55(5):644‐653. doi:10.1111/epi.12549 24621352

[37] Koehl M , Ladevèze E , Catania C , Cota D , Abrous DN . Inhibition of mTOR signaling by genetic removal of p70 S6 kinase 1 increases anxiety‐like behavior in mice. Transl Psychiatry. 2021;11(1):165. doi:10.1038/s41398-020-01187-5 33723223 PMC7960700

[38] Hadamitzky M , Herring A , Kirchhof J , et al. Repeated systemic treatment with rapamycin affects behavior and amygdala protein expression in rats. Int J Neuropsychopharmacol. 2018;21(6):592‐602. doi:10.1093/ijnp/pyy017 29462337 PMC6007742

[39] Rutishauser U , Ross IB , Mamelak AN , Schuman EM . Human memory strength is predicted by theta‐frequency phase‐locking of single neurons. Nature. 2010;464(7290):903‐907. doi:10.1038/nature08860 20336071

[40] Uhlhaas PJ , Roux F , Rodriguez E , Rotarska‐Jagiela A , Singer W . Neural synchrony and the development of cortical networks. Trends Cogn Sci. 2010;14(2):72‐80. doi:10.1016/j.tics.2009.12.002 20080054

[41] Cammalleri M , Lütjens R , Berton F , et al. Time‐restricted role for dendritic activation of the mTOR‐p70S6K pathway in the induction of late‐phase long‐term potentiation in the CA1. Proc Natl Acad Sci U S A. 2003;100(24):14368‐14373. doi:10.1073/pnas.2336098100 14623952 PMC283598

[42] Frey U , Morris RG . Synaptic tagging and long‐term potentiation. Nature. 1997;385(6616):533‐536. doi:10.1038/385533a0 9020359

[43] Atkins CM , Davare MA , Oh MC , Derkach V , Soderling TR . Bidirectional regulation of cytoplasmic polyadenylation element‐binding protein phosphorylation by Ca^2+^/calmodulin‐dependent protein kinase II and protein phosphatase 1 during hippocampal long‐term potentiation. J Neurosci. 2005;25(23):5604‐5610. doi:10.1523/JNEUROSCI.5051-04.2005 15944388 PMC6724975

[44] Giese KP , Fedorov NB , Filipkowski RK , Silva AJ . Autophosphorylation at Thr286 of the alpha calcium‐calmodulin kinase II in LTP and learning. Science. 1998;279(5352):870‐873. doi:10.1126/science.279.5352.870 9452388

[45] Bayer KU , Schulman H . CaM kinase: still inspiring at 40. Neuron. 2019;103(3):380‐394. doi:10.1016/j.neuron.2019.05.033 31394063 PMC6688632

[46] Antion MD , Merhav M , Hoeffer CA , et al. Removal of S6K1 and S6K2 leads to divergent alterations in learning, memory, and synaptic plasticity. Learn Mem. 2008;15(1):29‐38. doi:10.1101/lm.661908 18174371 PMC2170513

[47] Chiang GG , Abraham RT . Phosphorylation of mammalian target of rapamycin (mTOR) at Ser‐2448 is mediated by p70S6 kinase. J Biol Chem. 2005;280(27):25485‐25490. doi:10.1074/jbc.M501707200 15899889

[48] Antoch MP , Wrobel M , Gillard B , et al. Superior cancer preventive efficacy of low versus high dose of mTOR inhibitor in a mouse model of prostate cancer. Oncotarget. 2020;11(15):1373‐1387. doi:10.18632/oncotarget.27550 32341756 PMC7170500

[49] Hameed B , Terrault N . Emerging therapies for nonalcoholic fatty liver disease. Clin Liver Dis. 2016;20(2):365‐385. doi:10.1016/j.cld.2015.10.015 27063275

