# Supplementary material for: Pharmacological inhibition of S6K1 rescues synaptic deficits and attenuates seizures and depression in chronic epileptic rats
Source: CNS Neurosci Ther. 2023 Sep 22;30(3):e14475. doi: 10.1111/cns.14475 (PMC10945394; doi:10.1111/cns.14475)
Supplement: Supplementary file 1 — Appendix S1. [file CNS-30-e14475-s001.zip › cns14475-sup-0001-AppendixS1.pdf]

Supplemental Information

Pharmacological inhibition of S6K1 rescues synaptic deficits and attenuates seizures and depression in chronic epileptic rats

Yuying Zhang[†], Xiaojuan Cheng[†], Luyan Wu[†], Juan Li, Changyun Liu, Mingjia Wei, Chaofeng Zhu[1,2*], Huapin Huang[1,2*], Wanhui Lin[1,2*]

Supplemental Experimental Procedures

Establishment of the SE model in rat

As previously described, status epilepticus (SE) was induced by the administration of lithium-pilocarpine following our previous study[1, 2]. Briefly, Lithium chloride (127 mg/kg, i.p., Sigma-Aldrich, L9650, CA, USA) was injected 16-18 h prior to pilocarpine administration. Atropine sulfate (1 mg/kg, i.p., Sigma-Aldrich, PHR1379, CA, USA) was injected 30 min before pilocarpine administration to reduce the peripheral cholinergic effects of the pilocarpine. Repeated doses (≤3) of pilocarpine hydrochloride (30 mg/kg, i.p., Sigma-Aldrich, P6503, CA, USA) were given to the rat every 30 min until the emergence of SE (Stage IV-V on Racine's scale)[3]. More than 60 min after the onset of SE, diazepam (10 mg/kg, i.p.) was administered to terminate the ictal activity.

Hippocampal injection of PF-4708671

Operated rats for PF-4708671 (Selleck, S2163, CA, USA) injections were induced with 5% isoflurane anaesthesia (RWD Life Sciences, Shenzhen, Guangzhou, China) in 100% O2 for 2 minutes and then anaesthesia was maintained at isoflurane concentrations of 2% using a stereotaxic instrument (RWD Life Sciences, Shenzhen, Guangzhou, China). Briefly, PF-4708671 injections were performed as described[4]. One microlitre/liter/injection site of PF-4708671 was delivered to the CA1 area of the hippocampus using a 5-μl syringe (Hamilton, Reno, NV, USA, Model 75N). The

injection sites identified from the rat stereotaxic atlas of Paxinos and Watson were as follows: −3.0 mm/±2.2 mm, −4.0 mm/±2.2 mm, −5.0 mm/±2.2 mm, −3.0 mm/±3.0 mm, −4.0 mm/±3.0 mm, −5.0 mm/±3.0 mm (anterior-posterior direction from the bregma (AP)/ mediolateral direction from the bregma (ML)) and the dorsal-ventral direction from the brain surface (DV) for six stereotaxic injections is 3.0±0.2 mm. After at least a 7-day recovery period, animals were allowed to proceed to the next experiment.

Electrode implantation

Rats were mounted in a stereotaxic instrument under isoflurane anaesthesia as described previously. The hole was drilled first through the skull and EEG and EMG electrodes were implanted. An insulated tungsten wire (127μm in diameter; Cat No.796500; A-M Systems, Sequim, WA, USA) was implanted in the hippocampus CA1 region (AP: 3.0 mm, ML: 2.8 mm, DV: 3.0 mm, following Paxinos and Watson, 2005) for the assessment of EEG activity. Reference and ground stainless-steel screw electrodes were placed extra-axially overlying the cerebellum. Skeletal muscle activity (EMG) was monitored using two insulated silver wires (Cat No.786000; A-M Systems, Sequim, WA, USA) that were inserted bilaterally into the dorsal nuchal musculature. The assembly was fixed to the skull using dental cement. After surgery, rats were kept warm, received saline (0.9% NaCl, s.c.), and were housed individually after recovery from anaesthesia. Rats were allowed to recover for at least 10 days before their adaptation to the custom-made transparent cage (30 cm x 30 cm x 40 cm) and recording cable through a slip ring for one day.

Analysis of LFP data

As previously described[5], the power spectral density (PSD) values in 7 frequency bands were calculated by fast Fourier transform (FFT) using MATLAB software. (Math Works, Natick, MA, USA). To balance individual variability, we randomly selected three nonepileptiform discharge LFP segments as a baseline of approximately equal duration to the mean seizure episode duration for each rat in each group to normalize

the PSD values of LFP power in different frequency bands for each rat.

Behaviour Tests

Sucrose preference test (SPT)

As described previously[6], adaptation to sucrose drinking water before the experiment, after 24 h of water ban, each rat was given two new bottles, supplemented with 1% sucrose water and tap water, respectively. All rats had equal access to the two bottles. During the test, the positions of the two bottles were changed to avoid possible side preference effects, which was determined as side-preference (%) = sucrose water intake (g)/ (sucrose water intake (g) + tap water intake (g)) × 100%.

Forced swim test (FST)

The modified rat FST used was similar to the previously described protocol [7], and the depression levels of rats were measured with the forced swimming experiment video analysis system (SANS SA209; Saiangsi, Inc., China). Rats were individually placed in a Plexiglas cylinder (20 cm diameter, 40 cm height) containing 30 cm water (25 ± 1 °C) and were videotaped for 6 min. Time (sec) spent in passive (immobility) for the 2–6min was calculated for every rat and utilized as an index of depressive-like behaviour. After the swim session, Rats were dried and placed in a cage surrounded by a heating pad. The water was changed between each animal.

Tail suspension test (TST)

As described previously, Rats were hung uncontrollably by their tail for 6 min [8], and the depression levels of rats were measured with the tail suspension experiment video analysis system (SANS SA210; Saiangsi, Inc., China). Time (sec) spent in immobility for the last 4min was calculated for every rat and utilized as an index of depressive-like behaviour. Following the tail suspension, release the rat from the rack, gently remove the tape from the tail, and transfer it back to the cage for recovery.

Elevated plus maze (EPM)

As described previously[9], rats' anxiety levels were measured with the elevated plus maze experiment video analysis system (SANS SA211A; Saiangsi, Inc., China). The SANS animal behaviour software was used to trace and quantify rat movement in the unit for 5 min. The duration of time spent in open arms was used to calculate the extent of anxiety-like behaviour in each rat.

Protein extraction and Western blotting

Synaptosome protein samples were extracted following the manufacturer's protocols (Syn-PER™ Synaptic Protein Extraction Reagent, 87793; Thermo Fisher, USA).
Total protein samples were lysed in RIPA buffer (Sigma Aldrich) supplemented with protease inhibitors (Cat.No: HY-K0010, MCE, USA) and phosphatase inhibitors (Cat.No: HY-K0021, MCE, USA).
The protein concentration in the supernatant was determined using a BCA assay kit (P0010S, Enhanced BCA Protein Assay Kit; Beyotime). Equal amounts of protein samples were loaded and separated by 4-12% SDS-PAGE (GenScript ExpressPlus™ PAGE Gel, M00653) and transferred to PVDF membranes (Millipore, USA).
The membranes with synaptosome protein were incubated with antibodies including: CaMKII-α (1:1000, Mouse, 50049S; CST), Phospho-CaMKII (Thr286) (1:1000, Rabbit, 12716S; CST), NMDAR2B (GluN2B) (1:1000, Rabbit, 4207; CST), Phospho-S6 (Ser235/236) (1:1500, Rabbit, 4858; CST), Phospho-S6 (Ser240/244) (1:1500, Rabbit, 5364; CST), S6 (1:1000, Rabbit, 2217; CST), Phospho-mTOR (S2448) (1:1000, Rabbit, ab109268; Abcam), mTOR (1:2000, Rabbit, 28273-1-AP; Proteintech), GAPDH (1:1500, Rabbit, EM32010-02; EMAR).
Membranes were incubated with primary antibody overnight at 4 °C. After washing, the membranes were incubated with peroxidase-conjugated secondary antibodies (1:5000, goat anti-rabbit IgG, goat anti-mouse IgG, Abcam) for 1.5 h at room temperature. The immunoreactive bands were visualized by chemiluminescent HRP substrate (WesternBright ECL HRP Substrate Kit, K-12045-D50-EA, Advansta) with a

Fluorchem E Chemiluminescence Gel Imaging System (Protein Simple, USA). The intensity of the immunoreactive different exposure times for the same strip was determined with ImageJ software. The expression levels of the proteins of interest were examined as GAPDH/total protein in a semiquantitative manner.

Primary hippocampal neuronal cell culture

The hippocampal neuronal cell culture methods employed were similar to those described previously **[10, 11]**. In the present study, hippocampal neurons maintained in culture for 12 days were used for all experiments. Several reports showed that neurite injury was induced after 100-200μM kainic acid (KA) treatment **[10, 12]**, in this experiment all cultures were treated with 150 μM KA. Neurons were plated in 25 cm^2 cell culture flasks with a vented cap at 3.0 x 10^6 cells/flask and treated with indicated PF-4708671 or DMSO before the addition of KA. After 8 h of KA treatment, cultures were lysed in RIPA buffer and analyzed by SDS-PAGE followed by Western blotting as described above. Neurons were plated at ~0.10 x 10^6 cells/well in 48-well plates and 20 μM PF-4708671 was added to the cells 6 h before the addition of KA. Plates were fixed using 4% paraformaldehyde (PFA), immunostainedy using an mAb to β-tubulin(III) (1:2000, chicken, ab41489; Abcam), and imaged under an inverted fluorescence microscope (Eclipse Ti-U, Nikon).

Supplemental Figures

Figure S1 (related to Figure 1): The decrease in 235/36 phosphorylation was correlated with the induction of neurite outgrowth.

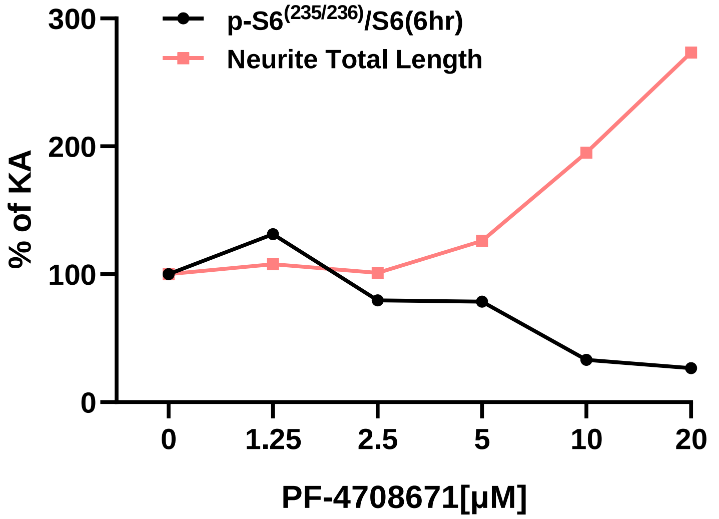

Pink lines represent the quantification of neurite total length of neurons treated with PF-4708671 expressed as a percentage of KA (DMSO). Black lines represent the quantification of phosphor S6 to pan S6 levels in 1A expressed as a percentage of KA (DMSO). Data are mean ± SEM.

Figure S2 (related to Figure 4): The postsynaptic potential amplitudes indicate the potentiation of synaptic transmission relative to the baseline.

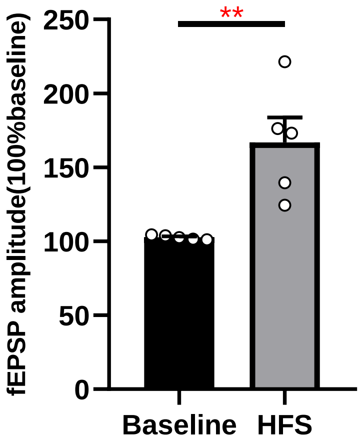

Bar histograms of normalized fEPSPs from the experiment in DMSO Control. A baseline was obtained and confirmed stable for 30 minutes before high-frequency stimulation (HFS) was applied. LTP amplitude was 166.9 % ± 16.78 % (relative to baseline) in DMSO controls (n=5). Data, mean ± SEM, *Unpaired t-test*. ***p* < 0.01.

Figure S3 (related to Figure 4): No significant difference was found in the in vivo CA3-CA1 LTP amplitude between the DMSO control and sham control groups

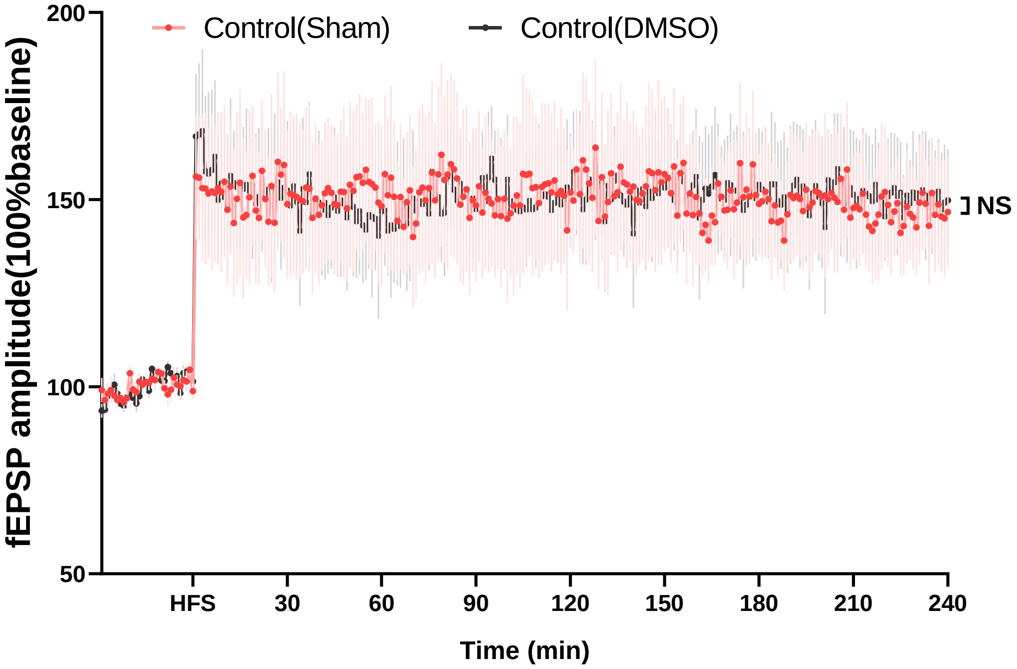

Summary plots of normalized fEPSPs in the anaesthetized rats under DMSO control (n = 5) and Sham Control (n = 6). Dots are the mean ± SEM of the normalized fEPSP amplitude for 1 min periods. Two-way ANOVA analysis of variance with repeated measures (Groups x Time) showed no significant differences between the sham control vs. DMSO control (P = 0.9924).

Figure S4 (related to Figure 5): Effect of PF-4708671 on the maturation of dendritic spines in the CA1 region of the hippocampus in chronic epileptic rats

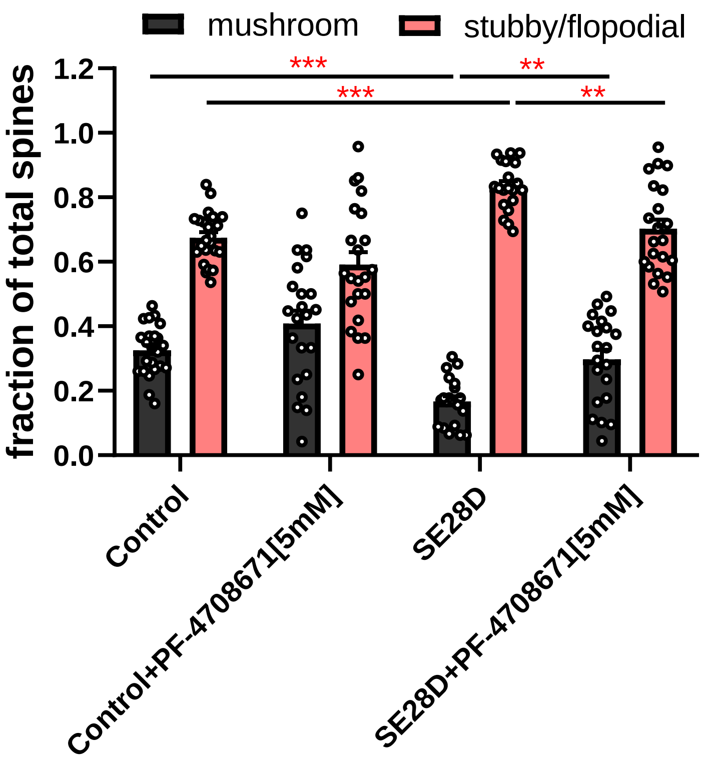

Spine Morphology Study Shows fractional stubby/filopodial and mushroom spines in CA1: Number of spines/10μm: n=20~23 dendrites (four or five neurons per rat, five rats), Scale bar =10μm. Data are plotted as mean ± SEM, $**p<0.01$, $***p<0.001$ by *two-way* ANOVA analysis with Tukey's multiple comparisons test.

References

1. Glien, M., et al., *Repeated low-dose treatment of rats with pilocarpine: low mortality but high proportion of rats developing epilepsy.* Epilepsy Res, 2001. **46**(2): p. 111-9 https://doi.org/10.1016/s0920-1211(01)00272-8.
2. Zhu, C., et al., *The Antagonism of 5-HT6 Receptor Attenuates Current-Induced Spikes and Improves Long-Term Potentiation via the Regulation of M-Currents in a Pilocarpine-Induced Epilepsy Model.* Front Pharmacol, 2020. **11**: p. 475 https://doi.org/10.3389/fphar.2020.00475.
3. Racine, R.J., *Modification of seizure activity by electrical stimulation. II. Motor seizure.* Electroencephalogr Clin Neurophysiol, 1972. **32**(3): p. 281-94 https://doi.org/10.1016/0013-4694(72)90177-0.
4. Al-Ali, H., et al., *The mTOR Substrate S6 Kinase 1 (S6K1) Is a Negative Regulator of Axon Regeneration and a Potential Drug Target for Central Nervous System Injury.* J Neurosci, 2017. **37**(30): p. 7079-7095 https://doi.org/10.1523/JNEUROSCI.0931-17.2017.
5. Li, D., et al., *Electrical stimulation of the endopiriform nucleus attenuates epilepsy in rats by network modulation.* Ann Clin Transl Neurol, 2020. **7**(12): p. 2356-2369

https://doi.org/10.1002/acn3.51214.

6. Guan, J., et al., *Early Life Stress Increases Brain Glutamate and Induces Neurobehavioral Manifestations in Rats.* ACS Chem Neurosci, 2020. **11**(24): p. 4169-4178 https://doi.org/10.1021/acschemneuro.0c00454.
7. Detke, M.J., M. Rickels, and I. Lucki, *Active behaviors in the rat forced swimming test differentially produced by serotonergic and noradrenergic antidepressants.* Psychopharmacology (Berl), 1995. **121**(1): p. 66-72 https://doi.org/10.1007/BF02245592.
8. Li, J. and D.J. Burgess, *Biomarker monitoring and long-acting insulin treatment in a stress model to facilitate personalized diabetic control.* J Control Release, 2021. **332**: p. 21-28 https://doi.org/10.1016/j.jconrel.2021.02.013.
9. Parihar, V.K., et al., *Predictable chronic mild stress improves mood, hippocampal neurogenesis and memory.* Mol Psychiatry, 2011. **16**(2): p. 171-83 https://doi.org/10.1038/mp.2009.130.
10. Xiang, Y., et al., *Inhibition of RhoA/Rho kinase signaling pathway by fasudil protects against kainic acid-induced neurite injury.* Brain Behav, 2021. **11**(8): p. e2266 https://doi.org/10.1002/brb3.2266.
11. Mattson, M.P., P. Dou, and S.B. Kater, *Outgrowth-regulating actions of glutamate in isolated hippocampal pyramidal neurons.* J Neurosci, 1988. **8**(6): p. 2087-100
12. Chong, P.N., et al., *Trkb-IP3 Pathway Mediating Neuroprotection in Rat Hippocampal Neuronal Cell Culture Following Induction of Kainic Acid.* Malays J Med Sci, 2018. **25**(6): p. 28-45 https://doi.org/10.21315/mjms2018.25.6.4.
