# Supplementary material for: Pharmacological inhibition of S6K1 rescues synaptic deficits and attenuates seizures and depression in chronic epileptic rats
Source: CNS Neurosci Ther. 2023 Sep 22;30(3):e14475. doi: 10.1111/cns.14475 (PMC10945394; doi:10.1111/cns.14475)

Full unedited blot for Figure 1A

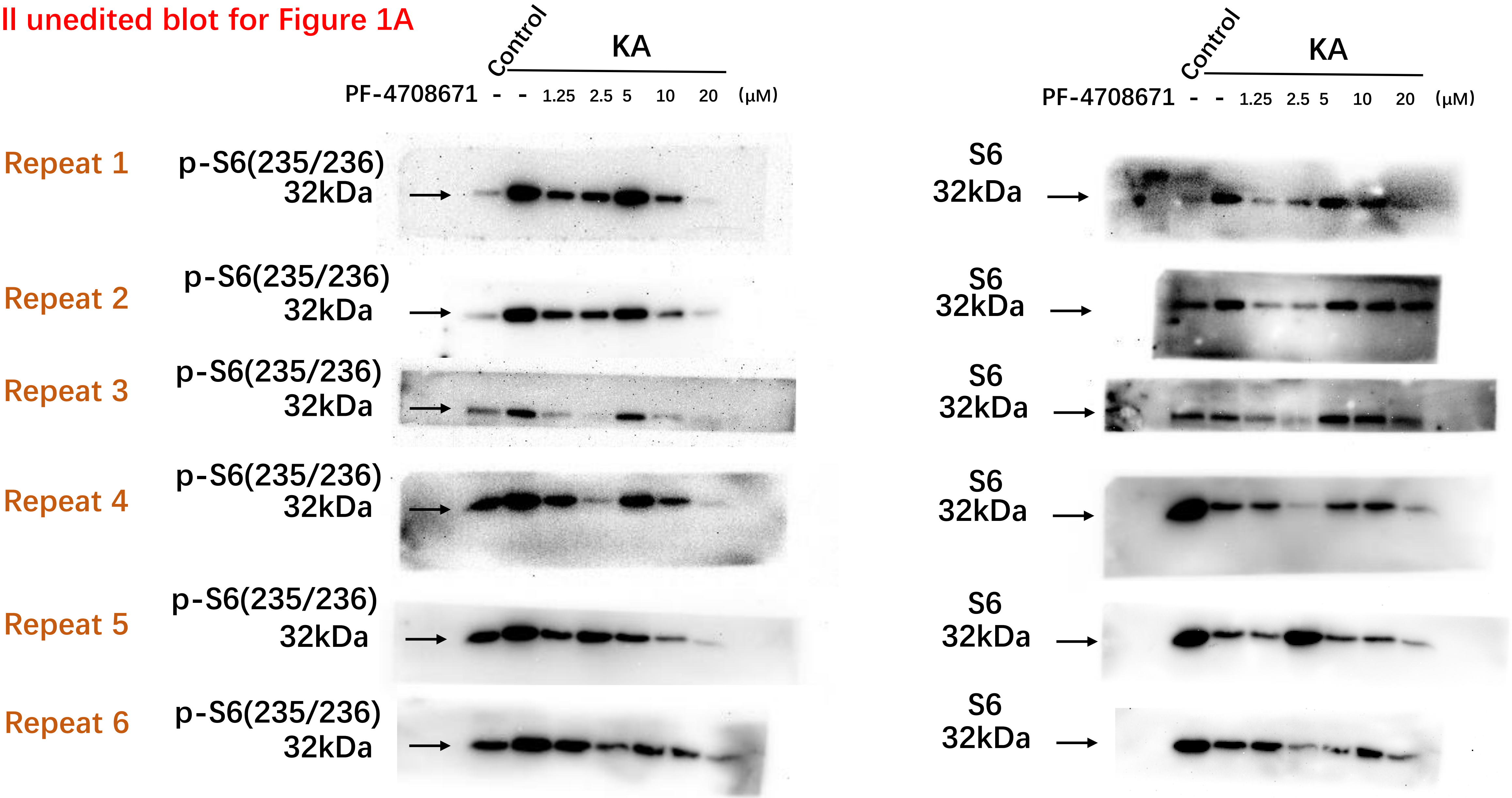

Membranes were incubated with phosphorylated antibodies（Left）, and total proteins antibodies（Right）were incubated on the same membranes after cleaning the primary antibody with western blot stripping buffer(21059; Thermo Fisher, USA).

Full unedited blot for Figure 1A

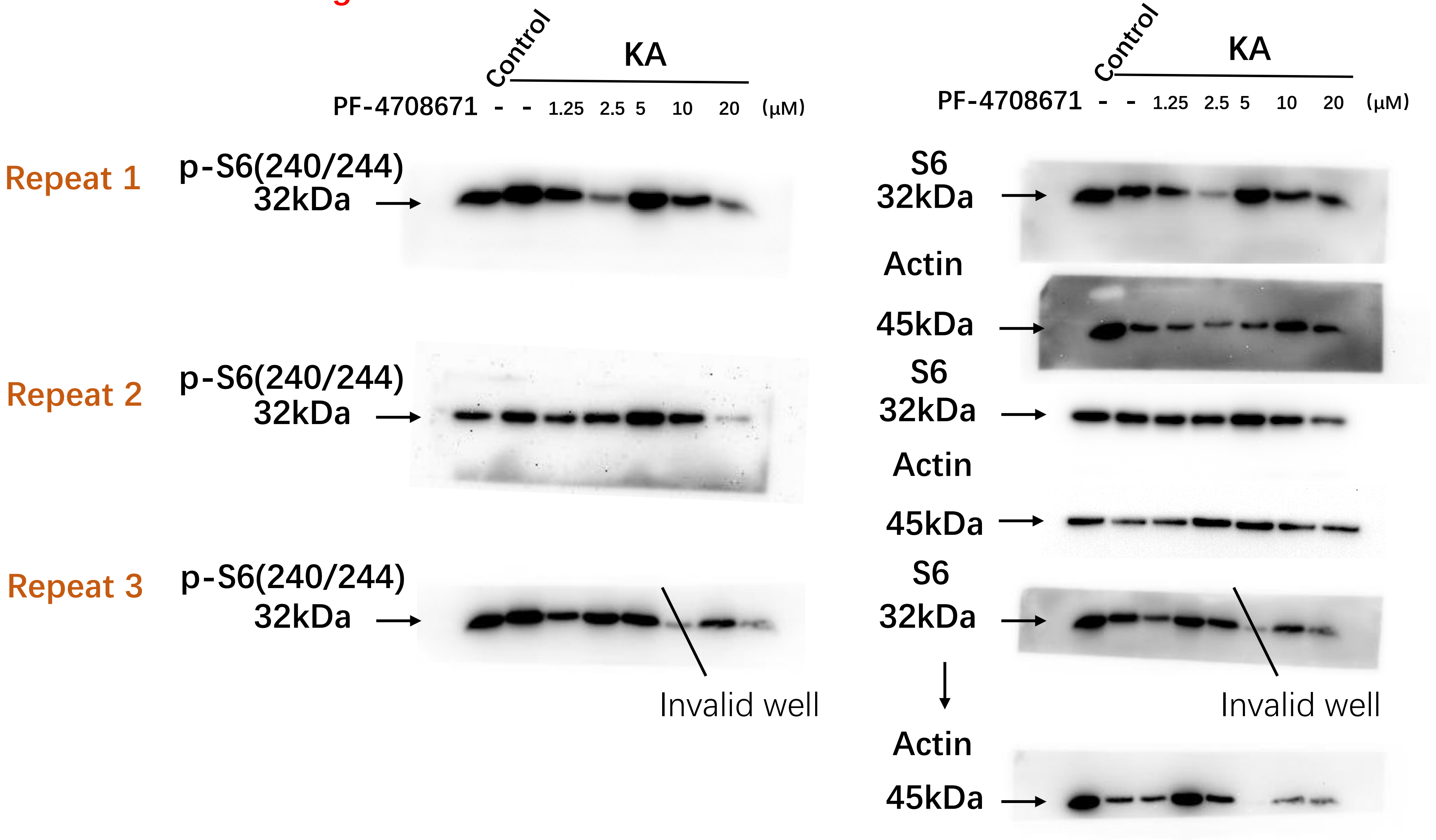

Membranes were incubated with phosphorylated antibodies (Left), and total proteins antibodies (Right) were incubated on the same membranes after cleaning the primary antibody with western blot stripping buffer(21059; Thermo Fisher, USA).

Full unedited blot for Figure 6 GluN2B

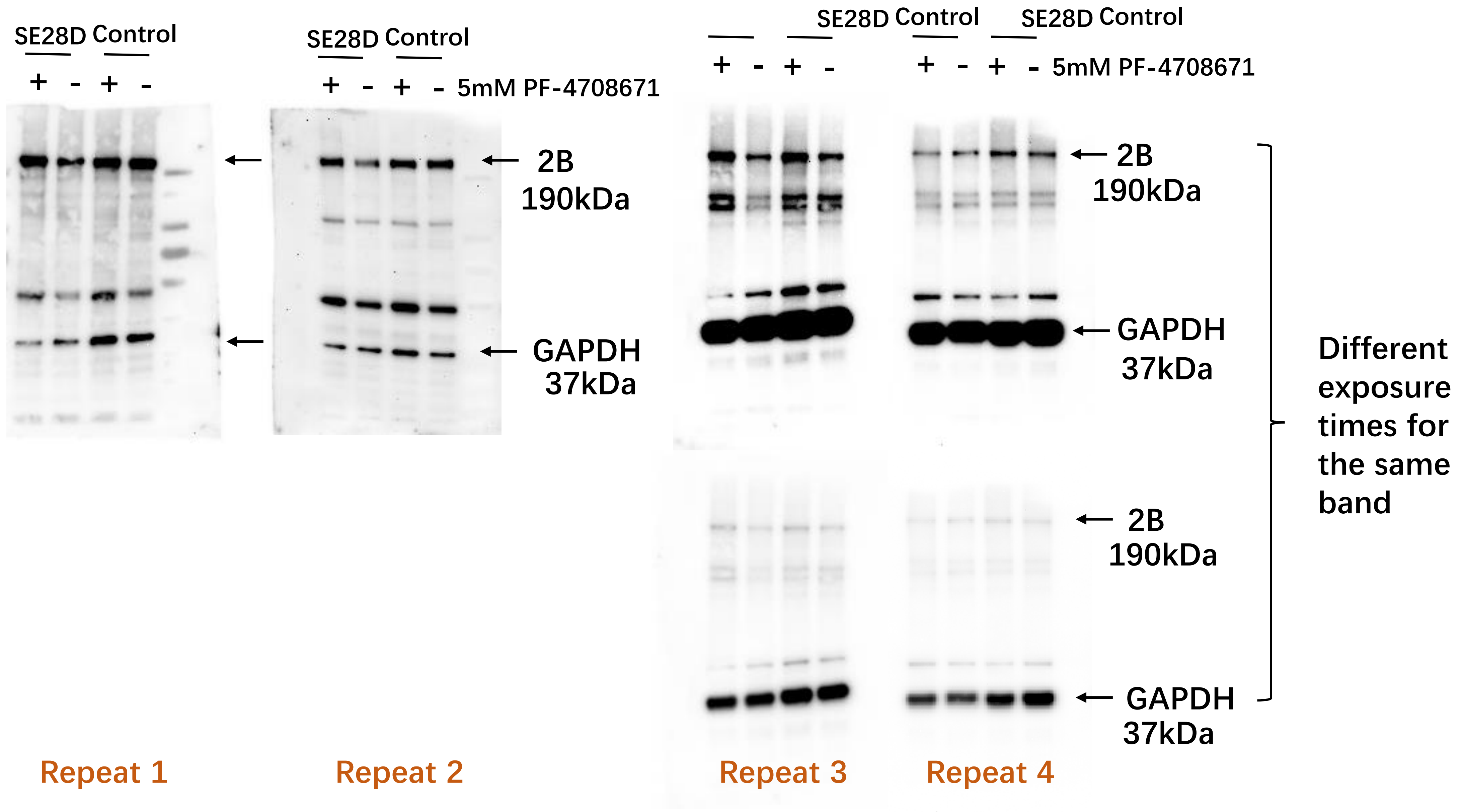

Full unedited blot for Figure 6 p-CaMKII-α(286)

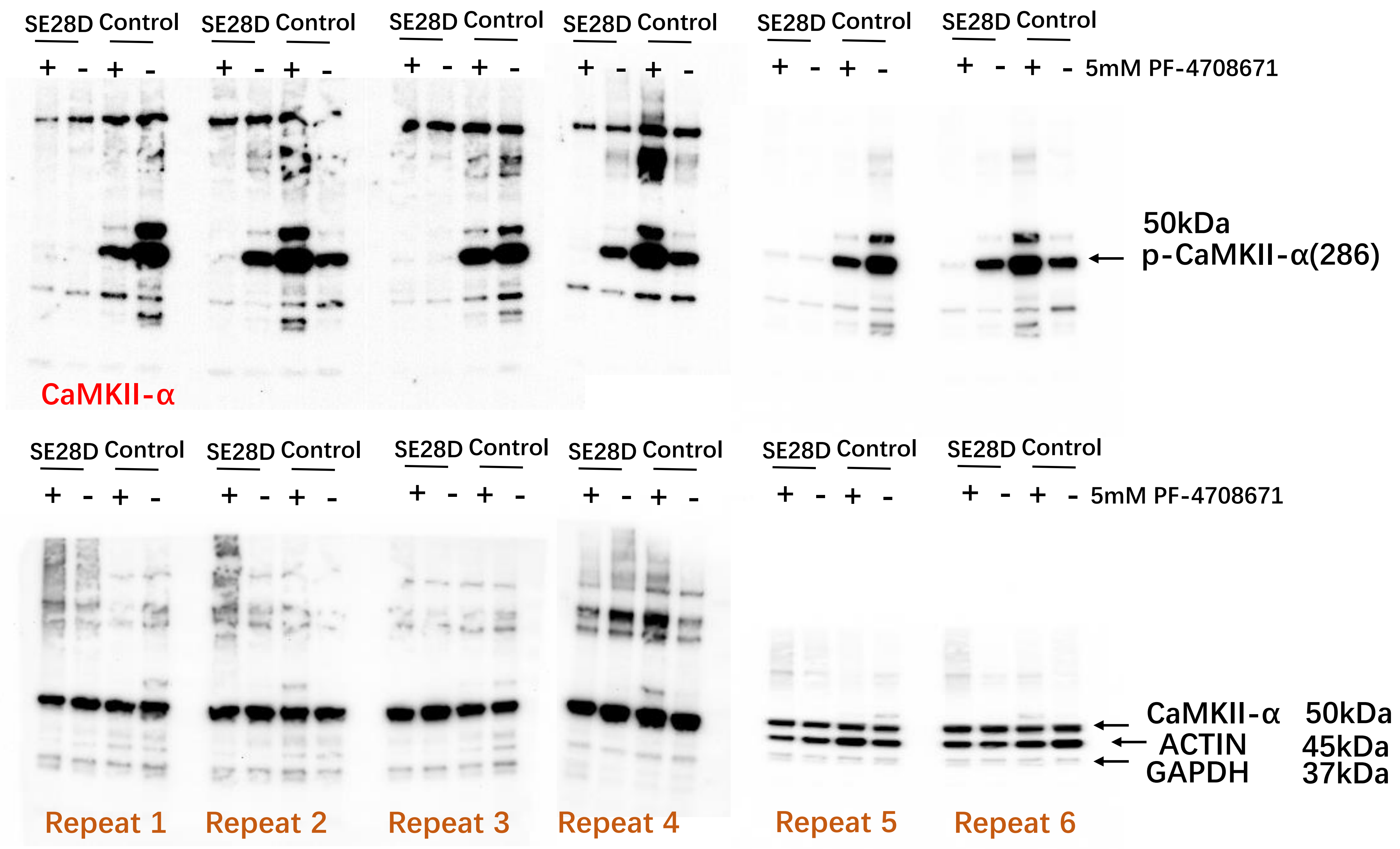

Full unedited blot for Figure 6 p-CaMKII-α(286)

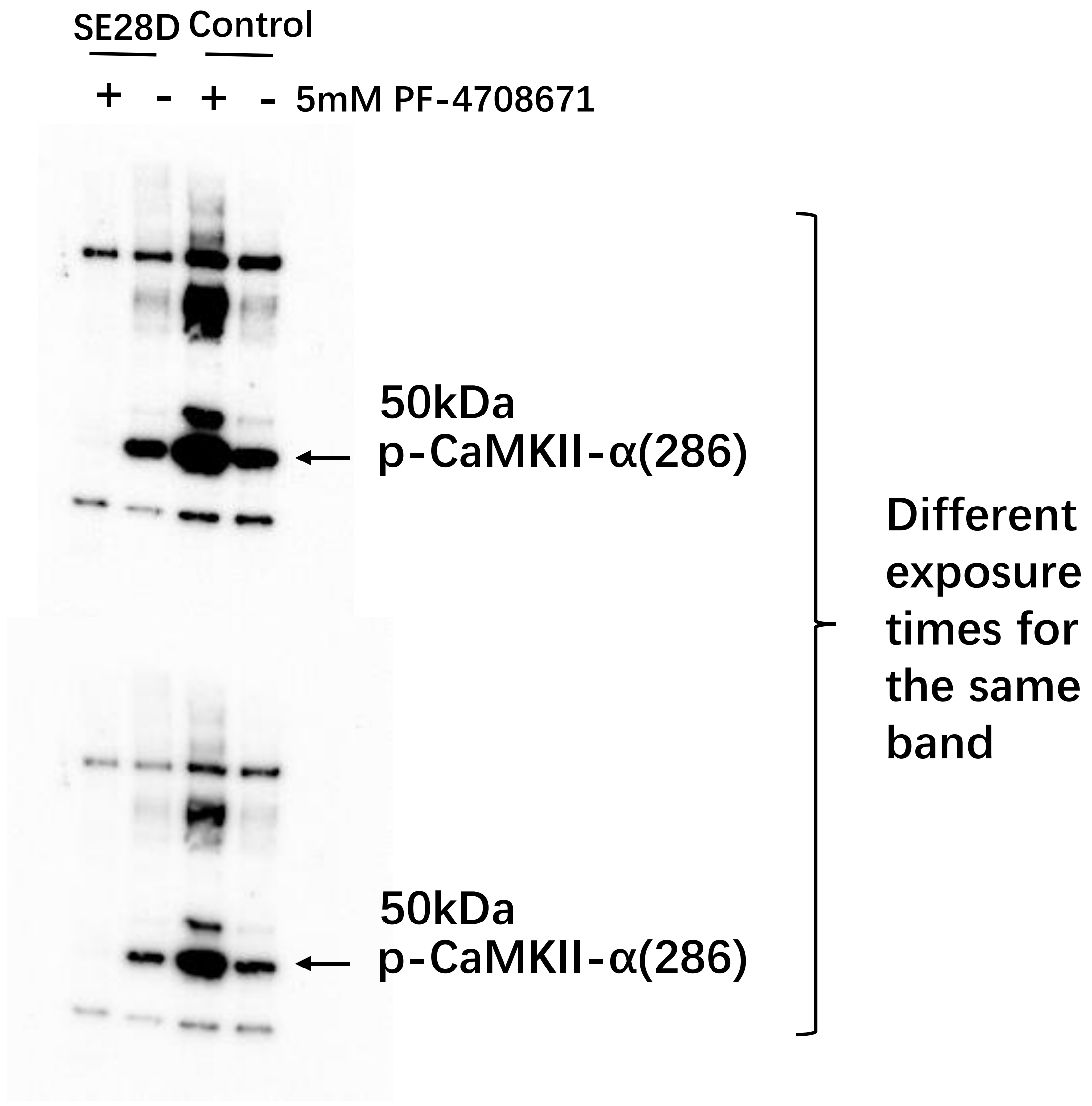

Repeat 4

Full unedited blot for Figure 6 CaMKII-α

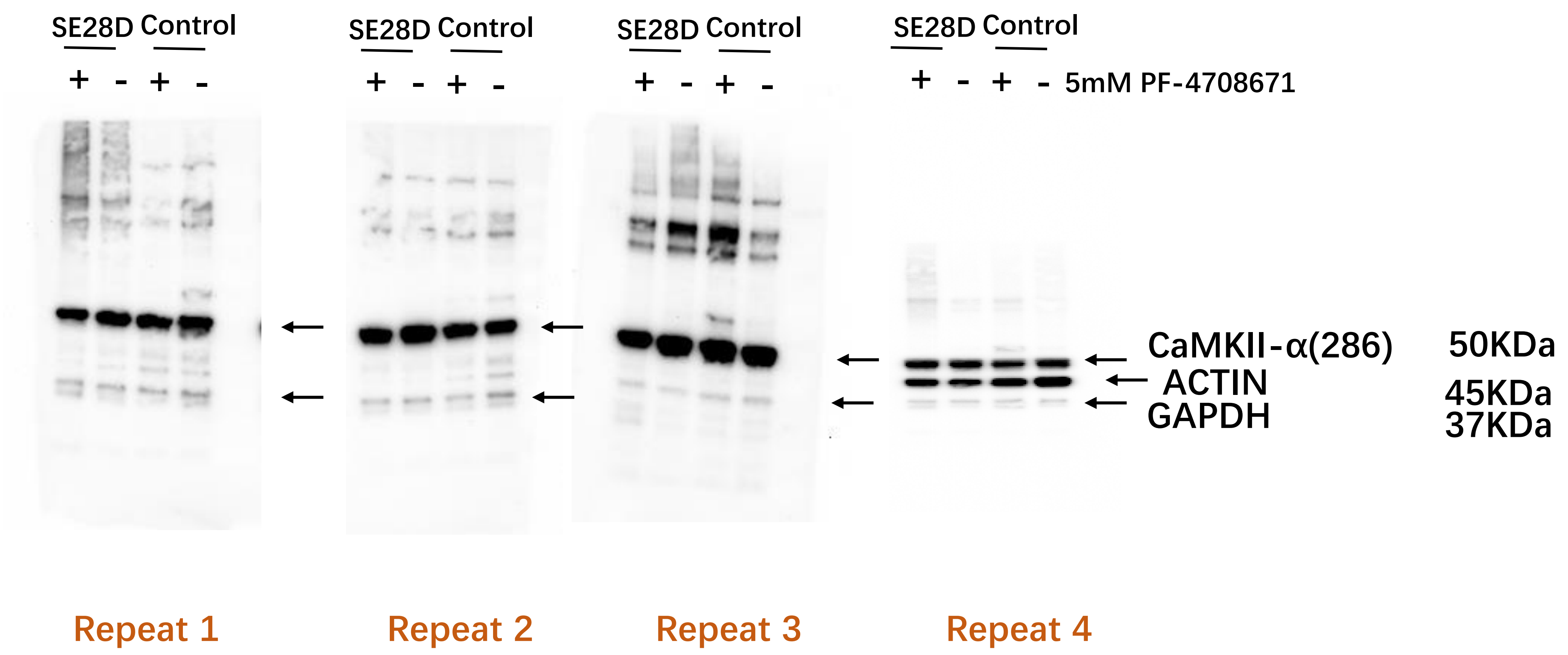

Full unedited blot for Figure 6 p-mTOR(2448)

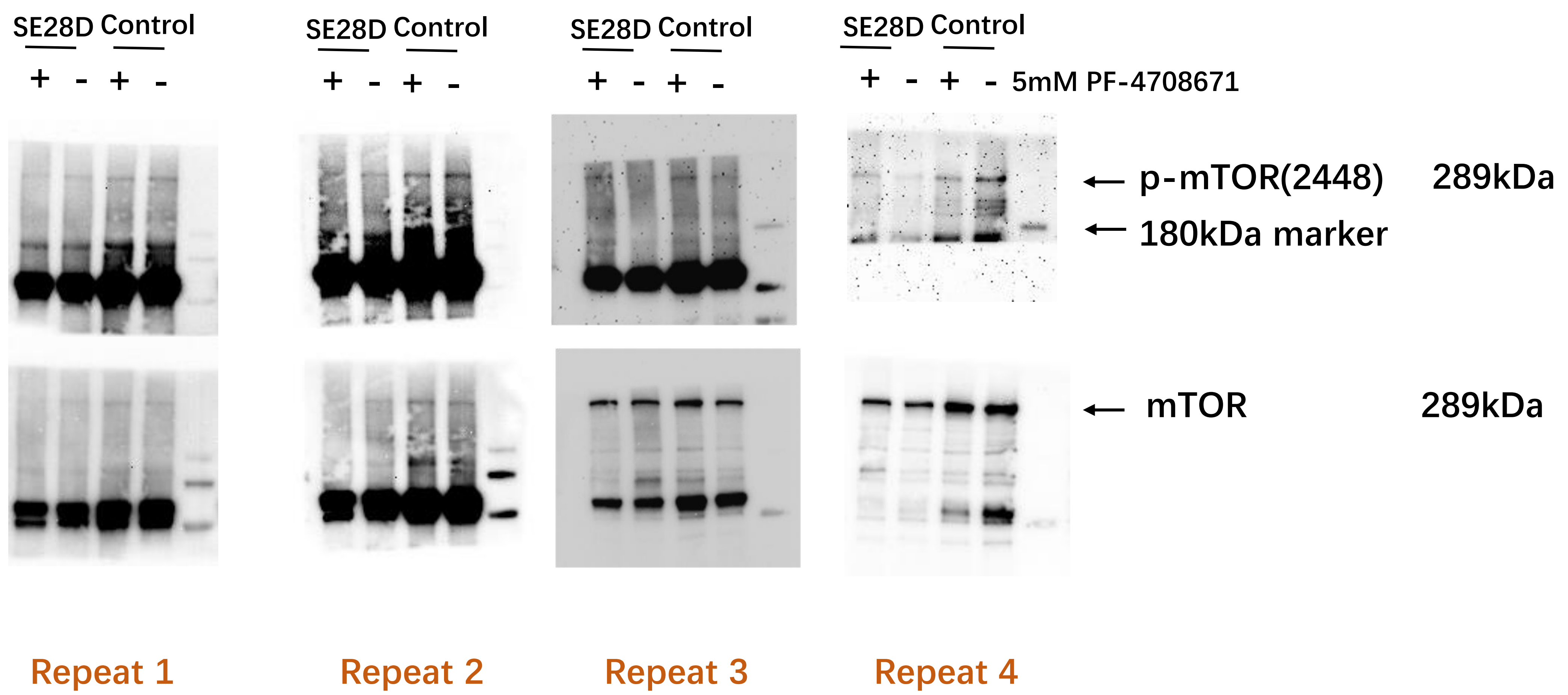

Full unedited blot for Figure 6 mTOR

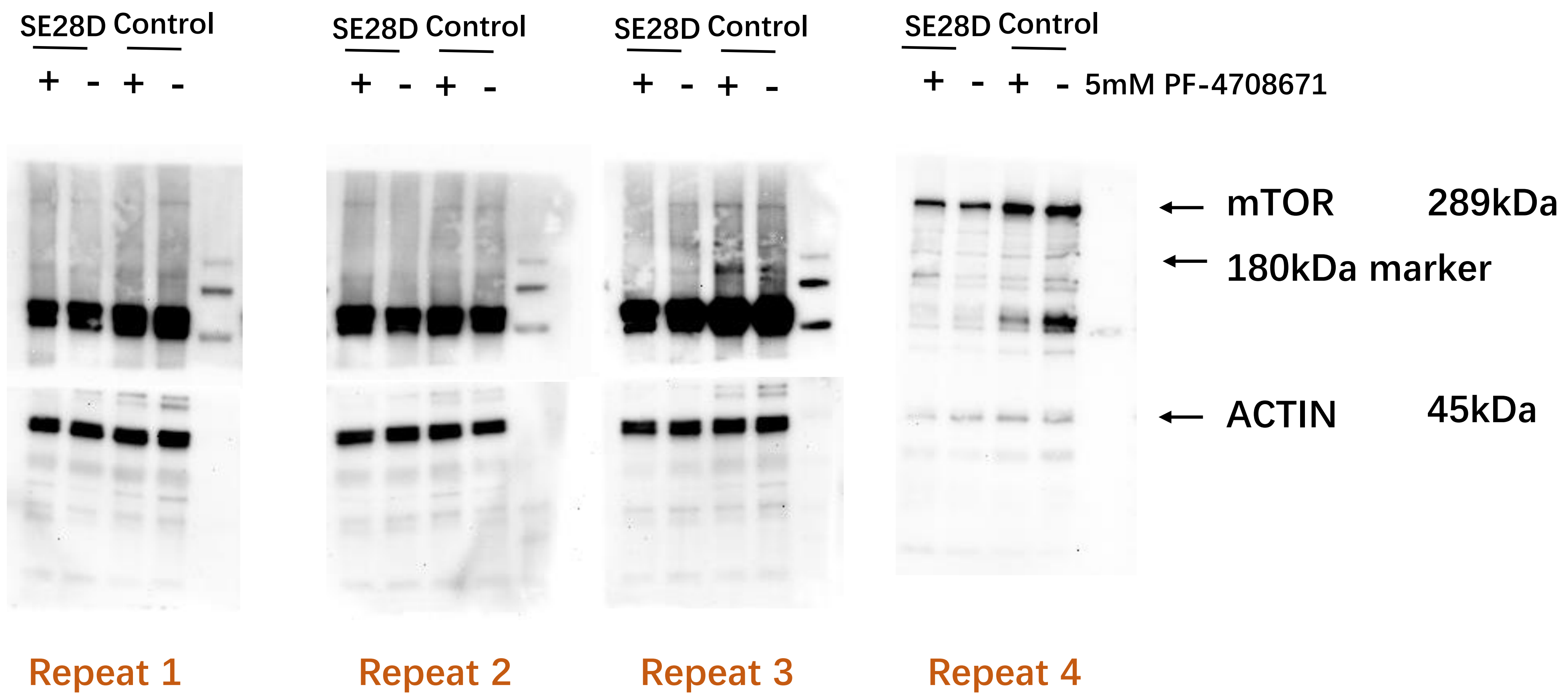

Supplement: Supplementary file 1 — Appendix S1. [file CNS-30-e14475-s001.zip › cns14475-sup-0002-Supinfo.pdf]
